# When copper turns killer: Decoding copper dyshomeostasis and cuproptosis in neurodegenerative pathogenesis and precision metal interventions

**DOI:** 10.4103/NRR.NRR-D-25-00808

**Published:** 2025-11-25

**Authors:** Wei Du, Tingyao Wu, Yonggang Fan, Min Zhao

**Affiliations:** 1 National Clinical Research Center for Laboratory Medicine, Department of Laboratory Medicine, The First Hospital of China Medical University, Shenyang, Liaoning Province, China; 2 Department of Hematology, The First Affiliated Hospital of Jinzhou Medical University, Jinzhou, Liaoning Province, China; 3 Key Laboratory of Brain Science Research Transformation in Tropical Environment of Hainan Province & Key Laboratory of Tropical Translational Medicine of Ministry of Education, College of Basic Medical Sciences, Hainan Medical University, Haikou, Hainan Province, China

**Keywords:** Alzheimer’s disease, amyotrophic lateral sclerosis, copper homeostasis, cuproptosis, Huntington’s disease, mitochondrial dysfunction, neurodegenerative disease, Parkinson’s disease, prion diseases

## Abstract

Copper is an essential cofactor for neuronal metabolism, enzymatic functions, and neurotransmission. However, copper dyshomeostasis-induced redox activity makes the brain vulnerable to oxidative and proteostatic stress. Cuproptosis, a recently characterized form of programmed cell death, is triggered by copper binding to lipoylated enzymes of the tricarboxylic acid cycle, resulting in proteotoxic stress, mitochondrial dysfunction, and cell death. Given that mitochondria are central to copper handling and the primary site of cuproptosis, we examine mitochondrial pathways and key cuproptosis-related genes. We also assess disease-specific signatures of copper imbalance. In Alzheimer’s disease, excess copper binds to amyloid-β, promoting aggregation and neurotoxicity. In Parkinson’s disease, copper-bound α-synuclein fosters aggregation, while copper-driven redox cycling elevates reactive oxygen species. Cuproptosis worsens mitochondrial vulnerability in Parkinson’s disease and impairs cellular stress responses in Huntington’s disease. In amyotrophic lateral sclerosis, superoxide dismutase 1-related defects compromise antioxidant defenses alongside copper-dependent mitochondrial dysfunction. In prion diseases, copper facilitates prion protein misfolding and toxicity. Across these disorders, common features include mitochondrial dysfunction and cuproptosis hallmarks—such as enhanced protein lipoylation, elevated reactive oxygen species, impaired electron transport chain activity, fragile Fe–S clusters, and increased reliance on the tricarboxylic acid cycle—which collectively increase neuronal susceptibility to copper dyshomeostasis. Clarifying and understanding the critical roles of copper metabolism not only elucidates the pathogenesis of neurodegenerative diseases but also offers alternative therapeutic strategies. This review uniquely integrates the mitochondria-centered cuproptosis axis with copper dyshomeostasis across Alzheimer’s disease, Parkinson’s disease, Huntington’s disease, amyotrophic lateral sclerosis, and prion diseases, mapping convergent vulnerabilities to mechanism-grounded interventions and outlining testable translational routes.

**Facts**

• Copper dyshomeostasis in neurodegenerative diseases: The review unifies copper transport and metabolism at both organismal and cellular levels—spanning CTR1, ATP7A/B, metallothioneins, mitochondrial carriers, and iron crosstalk—clarifying how dysregulation drives pathology in Alzheimer’s disease, Parkinson’s disease, Huntington’s disease, amyotrophic lateral sclerosis, and prion diseases.

• Cuproptosis as a convergent mechanism: By linking cuproptosis pathways and associated genes (such as *FDX1*, *LIAS*, *DLAT*, and *LIPT1*) to mitochondrial dysfunction, cuproptosis is established as a shared vulnerability state in neurodegenerative diseases, closely associated with protein lipoylation, fragile Fe–S clusters, reactive oxygen species, damage to the electron transport chain, and dependence on the tricarboxylic acid cycle.

• Therapeutic avenues based on potential mechanisms: The review connects the discovery of mechanisms with intervention strategies (such as copper chelation therapy, nanotechnology-enabled delivery, and CRISPR-Cas9 technology), emphasizing the feasibility and significance of clinical translation.

**Open questions**

• How does copper toxicity differentially impact specific subtypes of neurons and glial cells across various brain regions and stages of disease? Which biomarkers can clarify this *in vivo* cell specificity?

• What therapeutic strategies can precisely restore copper homeostasis in the brain without disrupting essential copper-dependent processes systemically?

• How do aging, genetic variants, and environmental exposures interact to modulate cuproptosis and copper toxicity networks? Additionally, can multi-omic profiling identify patient subsets that are most likely to benefit from targeted therapies?

## Introduction

Neurodegenerative diseases (NDDs) are characterized by a decline in cognitive abilities, damage to the central nervous system, neuroinflammation, and impaired movement. Generally, these diseases occur when nerve cells in the brain and peripheral nervous system lose their function and die. According to epidemiological studies, some of the most well-known NDDs that have been extensively studied include Alzheimer’s disease (AD), Parkinson’s disease (PD), Huntington’s disease (HD), amyotrophic lateral sclerosis (ALS), and prion diseases. Among these, AD is the most prevalent NDD (Gustavsson et al., 2023). Although the exact pathogenesis of NDDs is not yet fully understood, aging and genetic factors have been identified as significant contributors to these disorders. Furthermore, neuroinflammation, reactive oxygen species (ROS), and mitochondrial dysfunction are involved in neuronal damage and contribute to neurodegeneration (Jiang et al., 2025). Many studies have focused on deciphering the critical roles of metal ions in the pathophysiology of NDDs. Metals are essential for numerous critical processes involved in metabolism and biochemical pathways across all forms of life (Kostova, 2023). Copper (Cu), iron (Fe), zinc (Zn), and magnesium (Mg) play vital roles in brain biology and are essential for maintaining the functioning of the nervous system. However, while these metal ions are critical for biological processes, they can also be potentially hazardous to cells involved in redox reactions that lead to the accumulation of ROS, protein misfolding, and the progression of neurodegeneration. Thus, metal dysregulation is well understood as a critical inducer of several NDDs, including AD, PD, HD, and ALS (Chen et al., 2025).

Cu participates in numerous biochemical processes, including mitochondrial respiration, metabolism, signaling transduction, iron metabolism, and critical protein functions (Tsang et al., 2021). Maintaining Cu homeostasis is essential for cellular function. Both Cu deficiency and excess can lead to detrimental consequences. Under normal circumstances, Cu levels are kept relatively low, with the uptake, distribution, and elimination of Cu regulated by various enzymes and molecular pathways (Lutsenko et al., 2025). Cu ions can be obtained from our daily diet, including seafood, chocolate, and red wine. The small intestine enterocytes and duodenum are the primary sites for Cu absorption. Subsequently, this ion is either transported to organelles to perform its chemical functions or exported into the bloodstream. In humans, the liver and brain are the main storage organs for Cu, and the levels of Cu are tightly regulated in these two organs.

Recently, a novel programmed cell death pathway induced by excessive Cu in cells has been identified. This pathway is termed cuproptosis and is distinct from other known cell death pathways, such as apoptosis, ferroptosis, and necroptosis (Tsvetkov et al., 2022). The mechanism of cuproptosis is related to mitochondrial respiration and the α-lipoic acid (LA) pathway. During the cuproptosis process, Cu directly binds to lipoylated components of the tricarboxylic acid (TCA) cycle, leading to the aggregation of lipoylated proteins and the loss of iron-sulfur cluster proteins. This subsequently results in proteotoxic stress and cell death. In humans, it has been determined that genetic mutations causing intracellular Cu dysregulation lead to severe, life-threatening pathological conditions, such as Wilson’s disease and Menkes disease (Locatelli and Farina, 2025). The imbalance of Cu metabolism and cuproptosis has been implicated in the pathogenesis of neurodegeneration (Lane et al., 2025).

The excessive accumulation of Cu in amyloid plaques and Lewy bodies has been identified as a critical pathogenic hallmark in AD and PD, respectively. At the cellular level, Cu overload disrupts normal cellular processes by contributing to ROS generation, inducing oxidative stress, and subsequently causing damage to proteins, lipids, and DNA. Additionally, Cu -induced cuproptosis enhances the aggregation of amyloid-β proteins and α-synuclein, promoting the progression of AD and PD (Mangalmurti and Lukens, 2022). During the pathogenesis of HD, excessive Cu contributes to the generation of oxidative stress, leading to mitochondrial dysfunction. Abnormal levels of Cu have been found in the spinal cord and brain tissues of individuals with ALS, and this metal ion has been shown to cause the conversion of normal prion proteins (PrP) into structures associated with prion diseases (Locatelli and Farina, 2025). However, evidence to decipher the molecular mechanisms of Cu dysregulation and cuproptosis in the pathogenesis of these five neurodegenerative diseases is still lacking. Moreover, understanding the pathogenic roles of Cu and cuproptosis may provide therapeutic strategies for NDDs. Rebalancing Cu homeostasis and alleviating oxidative stress could potentially mitigate neuronal damage. Certainly, intensive studies and clinical trials are required to understand the mechanisms of Cu -induced neurotoxicity and to develop therapeutic strategies.

Numerous review articles have discussed the pathogenic roles of Cu and cuproptosis in NDDs; however, these articles have primarily focused on just one or two prevalent diseases, such as AD and PD (Huang et al., 2024; Jomova et al., 2025; Okafor et al., 2025). In this review, we highlight critical publications about the pathogenic roles of Cu and cuproptosis in AD, PD, HD, ALS, and prion diseases. Although relevant studies are not abundant and indicate a brand-new research field, we aim to provide a novel discussion that connects Cu and NDDs. Additionally, as the recently identified cell death pathway, cuproptosis-related research has drawn great attention in NDDs, and cuproptosis-related genes (CRGs) have been identified in several studies. This novel programmed cell death mechanism may offer a new perspective for understanding the pathogenic roles of Cu in NDDs. In this review, we summarize critical publications regarding these CRGs in different NDDs and provide new insights into cuproptosis and NDDs.

This review aims to synthesize and critically evaluate current published articles on Cu metabolism and its dysregulation, cuproptosis, and the pathogenesis of NDDs. It also seeks to clarify areas of consensus and controversy while identifying research gaps, thereby informing researchers about the latest progress in NDDs associated with this essential metal.

## Search Strategy

**Figure 1:**
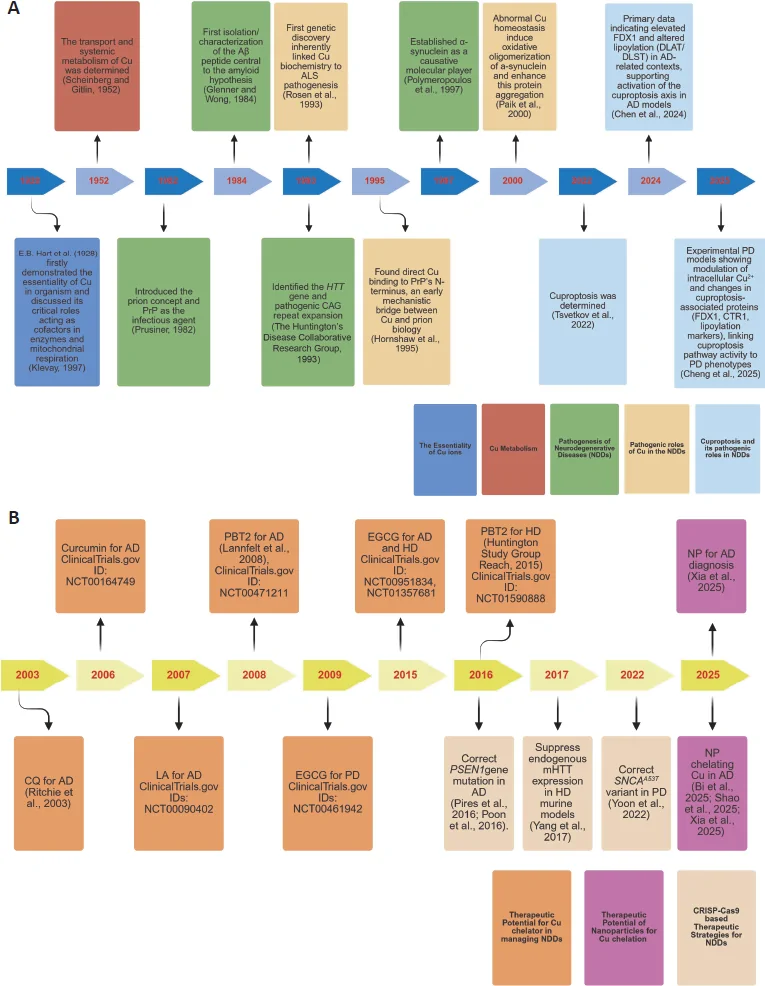
Timeline of major breakthroughs and paradigm-shifting findings regarding copper in NDDs. (A) A summary of key studies that have significantly advanced the understanding of Cu metabolism, the pathogenesis of neurodegenerative diseases, the pathogenic roles of Cu in these disorders, and cuproptosis from the early 1928 to the present. Created in BioRender. Du, W (2025) https://BioRender.com/enxotia. (B) Key studies and clinical trials conducted from 2003 to the present, focusing on the development of Cu chelators, nanoparticles, and CRISPR-Cas9-based gene editing tools for NDDs. Created in BioRender. Du, W. (2025) https://BioRender.com/d6y7ci3. AD: Alzheimer’s disease; ALS: amyotrophic lateral sclerosis; HD: Huntington’s disease; NDD: neurodegenerative diseases; NP: nanoparticles; PD: Parkinson’s disease.

To ensure a comprehensive literature review for our article focusing on Cu, cuproptosis, and NDDs, we conducted an extensive search of the PubMed and Web of Science databases for relevant peer-reviewed articles published up to June 2025. The search strategy used free-text keywords, including but not limited to: “copper,” “Cu,” “cuproptosis,” “copper-related programmed cell death,” “copper-related cell death,” “neurodegenerative disease,” “NDD,” “Alzheimer’s disease,” “AD,” “Alzheimer,” “Parkinson’s disease,” “PD,” “Parkinson,” “Huntington’s disease,” “HD,” “Huntington,” “amyotrophic lateral sclerosis,” “ALS,” “amyotrophic,” and “Prion disease.” Articles were included in this review if they met the following criteria: (1) they discussed the molecular mechanisms or roles of Cu and/or cuproptosis in the context of NDDs; (2) they provided experimental or clinical evidence, reviews, or meta-analyses; and (3) they were published in English in peer-reviewed journals. The exclusion criteria were as follows: (1) studies that did not primarily address Cu or cuproptosis in relation to neurodegeneration; (2) conference abstracts, editorial letters, theses, or non-peer-reviewed literature; (3) articles for which the full text was not available; and (4) studies that focused exclusively on non-neuronal diseases or other metals/metalloids without a significant discussion of Cu. The selection process was conducted independently by two reviewers, and any discrepancies were resolved through discussion to ensure objectivity and comprehensive inclusion of articles. The timelines and milestones of this review article are illustrated in **Figure 1**. These groundbreaking studies and research include Cu metabolism, the pathogenesis of NDDs, the pathogenic roles of Cu and cuproptosis in NDDs, and Cu and CRISPR-Cas9-based therapeutic strategies for NDDs. A total of 200 articles were cited in this review.

## Copper Homeostasis and Metabolism

### Copper metabolism in organisms

**Figure 2:**
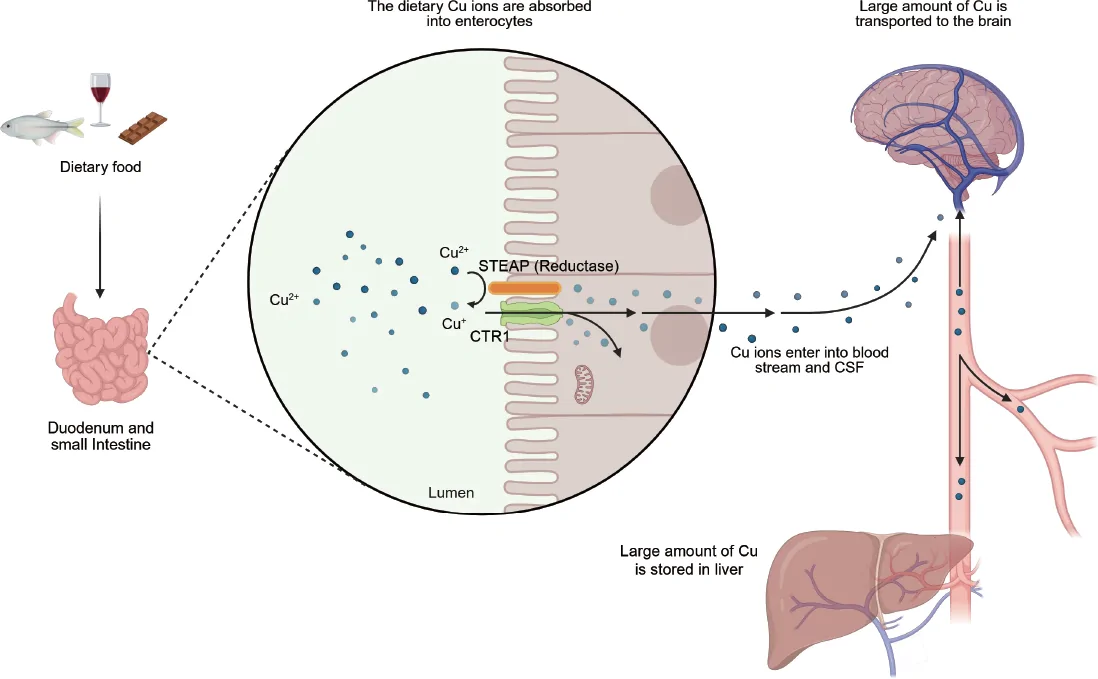
Abridged overview of Cu absorption in the human body. Cu-enriched foods, such as seafood, red wine, and chocolate, are consumed and digested in the human duodenum and small intestine. Cu^2+^ is reduced to Cu^+^ via the STEAP on the membrane of enterocytes, and subsequently, the Cu^+^ is transported into the cytoplasm through the CTR1. Large amounts of Cu are transported and stored in the liver and brain via the circulatory system to participate in physiological functions. Created in BioRender. Du, W. (2025) https://BioRender.com/iof6dbi. CSF: Cerebrospinal fluid; CTR1: Cu transporter protein 1; Cu: copper; STEAP: six-transmembrane epithelial antigen of the prostate.

**Figure 3:**
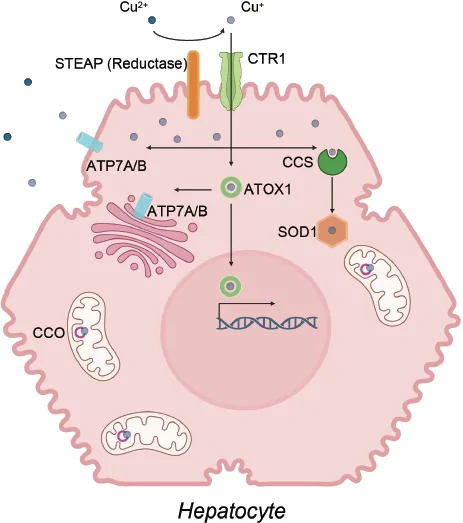
Diagram illustrating the pathways of cellular Cu metabolism in hepatocytes. Extracellular Cu^2+^ is reduced to Cu^+^ by STEAP and then transported into the cytoplasm by the Cu transporter protein, CTR1. Cu^+^ binds to Cu chaperones, including CCS and SOD1, and is then delivered to specific organelles, such as mitochondria, the Golgi apparatus, and the nucleus. In the Golgi apparatus, the Cu-ATPase transporters (ATP7A and ATP7B) transport Cu from the cytosol to the Golgi apparatus lumen, where they activate Cu-dependent enzymes. Cu is delivered by ATOX1 to the nucleus to regulate downstream gene expression. CCO binds to Cu and is involved in the electron transport chain in the inner mitochondrial membrane, where it reduces molecular oxygen and promotes ATP production. However, when the cytosolic Cu levels are high, ATP7A and ATP7B can export the Cu to the extracellular space. Created in BioRender. Du, W. (2025) https://BioRender.com/bt5iv75. ATOX1: Antioxidant 1 Cu chaperone; ATP7A: ATPase Cu transporter 7A; ATP7B: ATPase Cu transporter 7B; CCO: cytochrome c oxidase; CCS: Cu chaperone for superoxide dismutase; SOD1: superoxide dismutase 1.

The essential role of Cu has been established, and its critical functions as cofactors in metabolic enzymes and in regulating biological processes have been clearly outlined (Fu et al., 2025). Under normal circumstances, the concentration of Cu in serum ranges from 5 to 25 μM and from 70 to 80 μM in cerebrospinal fluid (CSF) for adults. Copper ions are not free or dissociated within the body; over 95% of these ions are bound to Cu chaperones (Wang et al., 2025d). The systemic levels of Cu are tightly regulated through a complex process that controls its transport, metabolism, elimination, and utilization. Initially, dietary Cu is primarily absorbed by Cu transporter 1 (CTR1), which is located in the enterocytes. Subsequently, the six-transmembrane epithelial antigen of the prostate (STEAP) and duodenal cytochrome b (DCYTB) proteins reduce Cu^2+^ to Cu^+^. The small intestine absorbs Cu ions, which are then secreted into the bloodstream and transported by several chaperones, such as albumin, transcuprein, histidines, and macroglobulins. The process of Cu uptake is illustrated in **Figure 2**. The liver serves as the primary organ for copper storage, and systemic copper is transported into hepatocytes via CTR1 (Teschke and Eickhoff, 2024). The intracellular Cu^+^ in hepatocytes is delivered to cytosolic Cu chaperones, including copper chaperone for superoxide dismutase (CCS) and superoxide dismutase 1 (SOD1), and is subsequently transported to specific cellular compartments, such as mitochondria, the Golgi apparatus, and the nucleus. In the mitochondria, cytochrome c oxidase (CCO) binds and delivers Cu to participate in the respiratory chain and redox pathways. Several Cu-dependent enzymes are activated in the secretory pathway of the Golgi apparatus. ATP7A and ATP7B, which are Cu^+^-ATPase transporters, play crucial roles in transporting copper from the cytoplasm to the lumen of the Golgi apparatus. They facilitate the exit of copper from this organelle when cytosolic copper levels rise. In addition to acting as enzymatic cofactors, copper ions can be transported by antioxidant 1 (ATOX1) and can directly bind to transcription factors to regulate downstream gene expression in the nucleus (**Figure 3**). ATP7A and ATP7B have distinct roles in copper metabolism: ATP7A transports copper outside the liver, while ATP7B excretes copper into bile. Dysfunction or depletion of these two proteins leads to copper accumulation and pathophysiological effects in the liver and brain. For instance, compromised ATP7A function can result in movement disorders, cognitive decline, and psychiatric manifestations (Aaltio et al., 2024), while malfunctions of ATP7B can cause hepatic toxicity, liver damage, inflammation, fibrosis, and cirrhosis due to impaired hepatic Cu transport (Sailer et al., 2024). In Wilson’s disease, mutations in the *ATP7B* gene that lead to dysfunction of this protein are critical factors in liver pathology (Shribman et al., 2021).

Cu is essential for central nervous system development, and the brain is another critical organ for Cu storage (An et al., 2022). The level of Cu in CSF is higher than in serum, and the entire human brain contains approximately 0.7–1.0 parts per million (ppm) of Cu; however, the concentration of Cu may vary depending on age, sex, and disease. The cerebellum, hippocampus, hypothalamus, olfactory bulb, and cortex contain relatively higher levels of Cu (Gromadzka et al., 2020). The blood–brain barrier (BBB) does not impede Cu from passing through; the uptake of Cu into neurons and glial cells is facilitated by the CTR1. Subsequently, intracellular Cu is bound and mediated by the CCS and ATOX1 (Xue et al., 2023). Similar to hepatocytes, neuronal homeostasis of Cu is coordinated by ATP7A and ATP7B for Cu transport and export among neurons (Wang et al., 2025d). Additionally, metallothioneins trap excessive Cu and play critical roles in neuroprotection by avoiding Cu-induced toxicity in the brain. Although Cu is recognized as essential for neural functions, the exact roles and molecular mechanisms of this metal ion require further elucidation through intensive studies.

### Copper metabolism in mitochondria

At the cellular level, Cu is critical for mediating mitochondrial function. Numerous mitochondrial enzymes require Cu as a cofactor to perform their biochemical functions. For example, the synthesis of cytochrome c oxidase 1 and 2 (SCO1 and SCO2), located in the inner membrane of mitochondria, is responsible for incorporating Cu ions into the catalytic sites of cytochrome c oxidase (COX), which is essential for its role in the electron transport chain (Povea-Cabello et al., 2024). However, excessive accumulation of Cu in the mitochondria can lead to significant cytotoxicity by disrupting respiration and inducing ROS production. It has been reported that increased levels of ROS, superoxide, and hydroxyl radicals are strongly associated with elevated levels of Cu ions in mitochondria (Vo et al., 2024). Similar to Fe, Cu induces ROS production and increases oxidative stress through the Fenton-Haber Weiss reaction: Cu^+^ + H_2_O_2_ → Cu^2+^ + OH^–^ + OH· and O_2_^–^ + H_2_O_2_ → O_2_ + OH^–^ + OH· (Li et al., 2023). Abnormal homeostasis and metabolism of Fe and Cu lead to distinct programmed cell death mechanisms referred to as ferroptosis and cuproptosis, respectively. While the detrimental effects of ROS production are central to both cell death mechanisms, the underlying processes are markedly different.

Moreover, dysregulation of iron has been shown to induce cell death through mechanisms distinct from apoptosis. This iron-induced cell death process is termed ferroptosis and is characterized by the accumulation of lipid peroxides within cellular membranes (Jiang et al., 2021). Several pathways involved in ferroptosis have been identified, including the guanosine triphosphate cyclohydrolase 1/tetrahydrobiopterin (GCH1/BH4) axis, the cysteine/glutathione/glutathione peroxidase 4 (GSH/GPX4) axis, and the ferroptosis suppressor protein 1/coenzyme Q (FSP1/CoQ) axis (Jiang et al., 2021). Fe dysregulation and Fe-induced ferroptosis are significant factors in the development of NDDs (Abdukarimov et al., 2025).

In 2021, a study demonstrated the association among ROS, ferroptosis, and the pathogenesis of AD. Several biomarkers of ROS and oxidative stress-induced lipid peroxidation, including NADPH oxidase 4 (NOX4), 4-hydroxynonenal (4-HNE), and malondialdehyde (MDA), were significantly elevated in impaired astrocytes of the cerebral cortex from patients with AD and AD animal models (specifically, the amyloid precursor protein [APP] and presenilin 1 [PS1] double transgenic mouse model). Overexpression of NOX4 continuously induces oxidative stress by promoting mitochondrial ROS generation, causing mitochondrial fragmentation, and inhibiting cellular antioxidant mechanisms. Furthermore, upregulation of NOX4 induces ferroptosis in human astrocytes. Thus, NOX4 promotes ferroptosis by inducing ROS-related lipid peroxidation and impairing mitochondrial metabolism, thereby exacerbating AD pathogenesis (Park et al., 2021). The same research team also elucidated the critical roles of NOX4 and ferroptosis in the pathogenesis of PD. They identified elevated levels of NOX4 in the hippocampus, accompanied by increased expression of myeloperoxidase and osteopontin. These two upregulated proteins induce mitochondrial dysfunction by impairing the mitochondrial electron transport chain and enhance ferroptosis by elevating levels of 4-hydroxy-2-nonenal (4-HNE), an end product of lipid peroxidation, in human astrocytes (Boonpraman et al., 2023).

**Figure 4:**
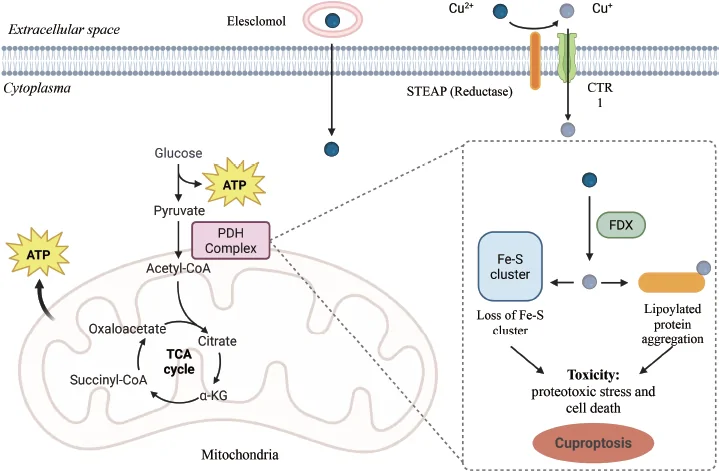
Schematic model of cuproptosis. Cu^2+^ can also be transported to the cytoplasm by the elesclomol, and then Cu ions are involved in the TCA cycle. FDX acts as the mitochondrial matrix reductase for Cu^2+^ reduction and serves as the upstream regulator of protein lipoylation. It facilitates the aggregation of mitochondrial proteins and the loss of Fe-S clusters. Subsequently, these aberrant processes lead to proteotoxic stress and cell death. Created in BioRender. Du, W. (2025) https://BioRender.com/6qzi17i. ATP: Adenosine triphosphate; CTR1: Cu transporter protein 1; FDX: Ferrdoxin; Fe-S: iron-sulfur; TCA: tricarboxylic acid; PDH: pyuvate dehydrogenase; STEAP: six-transmembrane epithelial antigen of the prostate.

Turning back to cuproptosis, several conflicting studies suggest that excessive Cu can induce cell death via distinct mechanisms, such as apoptosis, caspase-independent cell death, or the accumulation of ROS. However, a comprehensive understanding of cuproptosis in NDDs is still under discussion (Song et al., 2025). During the process of cuproptosis, the accumulation of lipoylated mitochondrial enzymes and the depletion of iron-sulfur (Fe-S) proteins have been observed. Additionally, mitochondria are the primary site of cellular apoptosis induced by Cu, while altered mitochondrial enzymes have been implicated in impairing the TCA cycle. Following treatment with a Cu ionophore, the analysis of cellular metabolites shows a time-dependent increase in the disruption of several metabolites associated with the TCA cycle. The inhibition of electron transport chain complexes I and II significantly reduces Cu-induced cell death, and exposure to Cu selectively modifies a group of metabolic enzymes through lipoylation, a widely conserved post-translational modification. Dihydrolipoamide S-acetyltransferase (DLAT), a lipoylated protein that is part of the pyruvate dehydrogenase complex, has been identified in this context (Rowland et al., 2018). Cu can bind directly to DLAT, promoting the aggregation of lipoylated DLAT through disulfide bonds. Moreover, the mitochondrial proteins ferredoxin (FDX1) and lipoyl synthase (LIAS) have been identified as important regulators of Cu toxicity (Tsvetkov et al., 2022; **Figure 4**). Deleting either the FDX1 or LIAS gene leads to an accumulation of pyruvate and α-ketoglutarate, inhibiting protein lipoylation and preventing cuproptosis. Furthermore, Cu toxicity disrupts the formation of Fe-S proteins by inhibiting the activity of mitochondrial assembly proteins (Du et al., 2023). These Fe-S proteins are essential cofactors for enzymes involved in the electron transport chain and various biochemical processes. Cu toxicity is a key factor contributing to the abnormal forms of Fe-S proteins, resulting in cell death.

Since the identification of cuproptosis by Tsvetkov et al. in 2022, numerous cuproptosis-related genes (CRGs) have been determined through various research efforts. These genes include *ATP7A*, *ATP7B*, *AGTR1*, *CDKN2A*, *DBT*, *DLD*, *DLAT*, *DLST*, *FDX1*, *GLS*, *GCSH*, *LIAS*, *LIPT1*, *LIPT2*, *MTF1*, *KIAA0319*, *NLRP3*, *NFE2L2*, *PDHA1*, *PDHB*, *SV2C*, *SLC6A3*, *SLC18A2*, and *SLC31A1* (Tsvetkov et al., 2022; Zhang et al., 2022a, 2023, 2024a; Wu et al., 2023; Ma et al., 2024). Although the specific pathogenic roles of these genes require further investigation, their expression patterns may serve as potential biomarkers for the diagnosis and prognosis of various diseases (Cai et al., 2025; Li et al., 2025; Liao et al., 2025; Liu et al., 2025c; Lyu et al., 2025; Xu et al., 2025a). In this review, we will discuss the role of CRGs in the pathogenesis of various NDDS in the subsequent sections.

## Copper Dysregulation and Cuproptosis in Neurodegenerative Diseases

Accumulating evidence indicates that both excessive and deficient Cu levels can result in serious pathophysiological conditions. Therefore, it is essential to acknowledge the connection between Cu homeostasis and NDDs.

### Alzheimer’s disease

AD is one of the most prevalent neurodegenerative disorders, identified over 100 years ago. It is characterized by impairments in memory, attention, language, and the ability to perform daily activities. There is a clear age-related correlation with AD, and the incidence of the disease is increasing in the elderly population (Liu et al., 2025b). For clinical diagnosis, two primary pathological hallmarks are associated with AD: the accumulation of amyloid-beta (Aβ) plaques and neurofibrillary tangles composed of hyperphosphorylated tau protein (Jia et al., 2025). Aβ is a peptide derived from the amyloid precursor protein (APP) and plays a significant role in the pathogenesis of AD through three interconnected processes. Aggregated Aβ peptides exhibit neurotoxic effects that lead to neuronal damage and cell death, primarily due to induced oxidative stress, inflammation, and excitotoxicity (Tolar et al., 2021). Furthermore, these abnormally structured Aβ peptides aggregate to form insoluble plaques that impede normal neuronal functions and contribute to synaptic dysfunction. The presence of abnormal Aβ peptides also triggers a neuroinflammatory response in microglia and astrocytes, which leads to chronic inflammation, further contributing to neurodegeneration and exacerbating the progression of AD (Zheng and Wang, 2025). Another critical pathological hallmark of AD is the formation of neurofibrillary tangles from hyperphosphorylated tau proteins. These microtubule-associated proteins are essential for stabilizing microtubules and facilitating intracellular transport in neurons. However, hyperphosphorylated tau proteins lose their ability to bind to microtubules, resulting in the destabilization of the microtubule network and impairing neuronal structure and function (Rawat et al., 2022). Additionally, the neurofibrillary tangles disrupt the cytoskeletal structure of neurons, impair normal biomolecule transport, and compromise cellular integrity. Consequently, the accumulation of these tangles leads to synapse loss and neuronal death, particularly in brain regions crucial for memory and cognition (Dong et al., 2022). In the pathogenesis of AD, aggregated Aβ peptides and hyperphosphorylated tau proteins interact in a mutually reinforcing manner. The accumulation of aggregated Aβ peptides is believed to be the initiating step that triggers a cascade of events leading to tau hyperphosphorylation and the formation of neurofibrillary tangles. In turn, the pathogenic tau proteins exacerbate Aβ neurotoxicity. Therefore, understanding the pathogenic roles of Aβ and tau proteins in AD is critical for the development of therapeutic interventions (Liu et al., 2024).

Disruptions in Cu homeostasis have been implicated in the pathogenesis of AD. Scolari Grotto and Glaser (2024) have found that serum Cu levels in patients with AD are higher compared to healthy controls. Furthermore, serum Cu levels have been positively correlated with aging (Wang et al., 2025c). In addition to clinical samples from patients with AD, animal models have played a crucial role in elucidating the importance of Cu in the disease’s pathogenesis. Notable examples include APP/PS1 and 5×FAD mice. The Cu levels in the brains of these models vary based on the specific characteristics of each mouse model and the stage of disease progression. APP/PS1 mice are transgenic models that express mutated forms of both amyloid APP and PS1, leading to the development of Aβ plaques similar to those found in AD. Abnormal Cu levels have been reported in these mice, with alterations in Cu homeostasis contributing to oxidative stress and neurotoxicity (Hosseinpour Mashkani et al., 2023). Similarly, 5×FAD mice express five familial AD mutations, resulting in accelerated Aβ plaque formation. Studies have indicated that Cu dysregulation in these mice may also contribute to Aβ aggregation and neurotoxicity (Solovyev et al., 2021; Xiao et al., 2025). Although the molecular mechanisms linking Cu dysregulation to tauopathies in another AD-related mouse model, P301S transgenic mice, are still not fully understood, several studies have highlighted the beneficial effects of LA in reducing oxidative stress, inflammation, and tauopathy induced by copper or iron overload (Zhang et al., 2024b; Manavi et al., 2025). LA acts as a critical cofactor for mitochondrial dehydrogenases and is essential for cellular antioxidant defense mechanisms and mitochondrial function (Kabin et al., 2023). *In vitro* experiments have shown that LA can bind to Cu and Fe, serving as a therapeutic agent to mitigate the consequences of Cu overload by upregulating selenoproteins and rebalancing redox reactions (Kabin et al., 2023). Importantly, the beneficial effects of LA for patients with AD have also been studied in clinical trials (ClinicalTrials.gov IDs: NCT01058941 and NCT00090402) (Pei et al., 2023; Kirss et al., 2024). Additionally, one research group utilized SH-SY5Y cell lines and a *Drosophila melanogaster* model of AD to investigate the therapeutic potential of LA, demonstrating that this cofactor can rebalance Cu homeostasis and alleviate symptoms (Metsla et al., 2022). Thus, further studies are needed to clarify the exact association between LA-sequestered Cu and AD pathogenesis. The development of specific and accurate AD mouse models that effectively replicate the disease’s pathogenesis in humans remains an active area of research.

**Figure 5:**
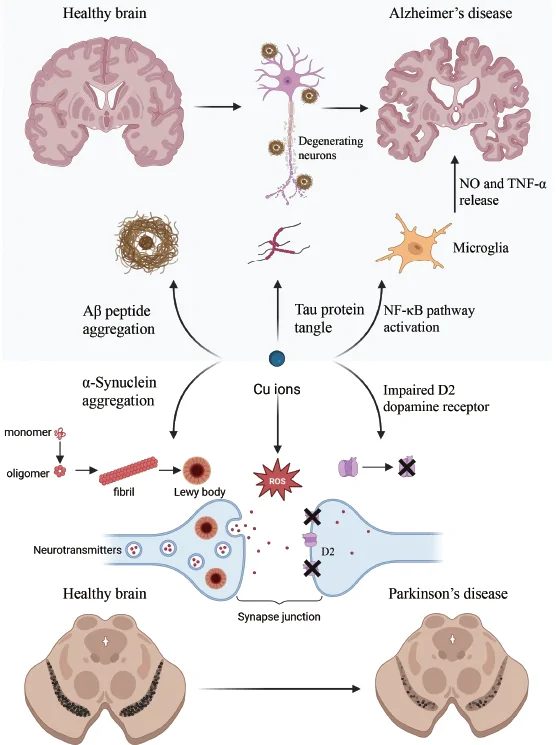
A diagram illustrating the pathogenic roles of Cu in AD and PD. Cu plays a critical role in the pathogenesis of AD and PD. The upper part of this figure displays the human brain tissue with the coronal cut at the basal ganglia. For the AD (upper part of this figure), Cu binds to the Aβ peptides and enhances the aggregation of Aβ. Cu can also induce the phosphorylation and tangle formation of tau protein. The last mechanism of Cu in the pathogenesis of AD is the activation of microglia through the NF-κB signaling pathway, resulting in the release of inflammatory factors, such as NO and TNF-α. The bottom half of this figure displays the human midbrain tissue at the superior colliculus. Cu enhances the pathogenesis of PD through three main mechanisms (the bottom half of this figure), including promoting α-synuclein aggregation, ROS generation, and impaired D2 dopamine receptors. Created in BioRender. Du, W. (2025) https://BioRender.com/w3xs87e. AD: Alzheimer’s disease; Aβ: amyloid-β; Cu: copper; NF-κB: nuclear factor kappa-B; NO: nitric oxide; PD: Parkinson’s disease; ROS: reactive oxygen species; TNF-α: tumor necrosis factor-α.

To date, three mechanisms have linked Cu to the pathogenesis of AD, as illustrated in **Figure 5**. First, excessive Cu binds to Aβ peptides, enhancing their aggregation and increasing neurotoxicity (Feng et al., 2023). Additionally, Cu ions bound to Aβ peptides significantly decrease the clearance of Aβ due to the formation of Cu-Aβ complexes, which reduce the expression of lipoprotein receptor-related protein 1. This reduction leads to increased accumulation of Aβ in the brain (Okafor et al., 2025). Second, nuclear factor kappa-light-chain-enhancer of activated B cells (NF-κB) signaling pathways, inducing the release of inflammatory factors such as nitric oxide (NO) and tumor necrosis factor-alpha (TNF-α) in microglia, which exacerbates neurodegeneration (Zhou et al., 2022). Lastly, Cu may promote the phosphorylation and aggregation of tau proteins, contributing to neurotoxicity (Okafor et al., 2025). Moreover, several clinical trials and animal studies examining the therapeutic potential of Cu chelators have highlighted the pathogenic roles of this transition metal in the progression of AD (Behar and Maayan, 2025; Lopez-Guerrero et al., 2025). The details of these studies and clinical trials will be discussed in Section “Copper chelators for the treatment of neurodegenerative diseases” (**Additional Table 1** and **Box 1**).

**Additional Table 1:** Preclinical investigations of Cu chelators for the treatment of neurodegenerative diseases

Chelator	Disease	Principle	Experiment	Reference
CQ	AD	Reduce Aβ aggregation and toxicity; improve cognitive function	Rat cortical primary neurons; APP/PS1 transgenic mice model	Cherny et al., 2001; Jenagaratnam and McShane, 2006; Bush, 2008; Wang et al., 2012
CQ	HD	Reduce the expression of mutant HTT proteins; alleviate symptoms in the HD mouse model, including improved behaviors, reduced accumulation of aggregated HTT, decreased insulin resistance, and diminished striatal atrophy	PC-12 cell lines; R6/2 mouse model	Nguyen et al., 2005
PBT2	AD	Improve cognitive function; reduce levels of Aβ_1-42_ in patients with AD; improve cognitive function; reduce excitotoxicity	Mouse primary neurons	Johanssen et al., 2015
PBT2	AD	Inhibit Aβ aggregation; mitochondrial protection; antioxidant; decrease apoptosis; decrease expression of cytokines; neuroinflammation inhibition via endoplasmic reticulum suppression	APP/PS1 transgenic mice model; 5xFAD mice model; SH-SY5Y, MES23.5 cell lines; APoEe4 transgenic mice model	Cho et al., 2007; Wang et al., 2009, 2014; Jaisin et al., 2011; Zheng et al., 2017; Kou et al., 2021
EGCG	AD	Reduce toxicity of Aβ aggregation; reduce Aβ-induced neurotoxicity and mitochondrial damage; reduce inflammation in microglia; inhibit NLRP3 inflammasomes and caspase 11-dependent inflammasomes; reduce mitochondrial dysfunction and ROS generation	Primary microglia; APP/PS1 transgenic mouse model	Zhong et al., 2019; Tavanti et al., 2020
LA	AD	Attenuate tau protein phosphorylation; improve cognitive function	P301S transgenic mice	Zhang et al., 2018
LA	AD	Normalize Cu metabolism in cell and fruit fly of AD; slightly weakens Cu- induced smooth eye phenotype	SH-SY5Y; *Drosophila melanogaster*	Metsla et al., 2022
LA	PD	Reduce neuroinflammation by inhibiting expression of NF-kB, TNF-α, and iNOS	Parkinsonian mouse model	Zhang et al., 2022b
	AD	Reduce expression of iNOS and TNF-α; suppress upregulation of NF-kB	APP/PS1 transgenic mice model; LPS- treated BV-2 cell line	Wang et al., 2018
TM	HD	Reduce striatal degeneration	R6/2 transgenic mice model	Tallaksen-Greene et al., 2009

AD: Alzheimer's disease; APP/PS1: Aβ: amyloid-beta; CQ: clioquinol; Cu: copper; EGCG: epigallocatechin gallate; HD: Huntington's disease; HTT: Huntingtin; LA: α-lipoic acid; LPS: lipopolysaccharide; NF-kB; nuclear factor kappa-light-chain-enhancer of activated B cells; PBT2: 5,7-dichloro-2-[(dimethylamino) methyl]-8-hydroxyquinoline; PD: Parkinson's disease; ROS: reactive oxygen species; TM: tetrathiomolybdate; TNF-α: tumor necrosis factor-alpha.

**Box 1:** Overall research design and the significance of clinical trials targeting copper dysregulation in neurodegenerative diseases

**• Aims of related clinical trials**
- These trials aim to evaluate whether restoring copper homeostasis can mitigate neurodegeneration by reducing amyloid burden, enhancing synaptic function, improving cognitive performance, and slowing clinical decline. - The interventions encompass metal-protein attenuating compounds, such as clioquinol and 5,7-dichloro-2-[(dimethylamino) methyl]-8-hydroxyquinoline, as well as natural copper chelators, including epigallocatechin gallate, curcumin, and α-lipoic acid.
**• Study designs**
-Predominantly Phase II randomized, double-blind, placebo-controlled studies were conducted across multiple countries, including Australia, Sweden, Germany, Spain, the United States, China, and India. -Sample sizes ranged from small early-phase cohorts (approximately 20 to 80 participants) to larger exploratory studies (approximately 100 to 480 participants), reflecting feasibility testing and biomarker-driven endpoints.
**• Study populations**
- Focus on Alzheimer’s disease, Huntington’s disease, and Parkinson’s disease, enabling crossdisease exploration of copperrelated mechanisms.
**• Key endpoints**
- Biomarkers included reductions in plasma and cerebrospinal fluid Aβ42 with clioquinol and 5,7-dichloro-2-[(dimethylamino) methyl]-8-hydroxyquinoline, respectively; modulation of inflammation and oxidative stress with alpha-lipoic acid; and imaging/connectivity endpoints with epigallocatechin gallate. - Clinical outcomes: exploratory effects on cognition (Alzheimer’s disease) and motor function (Huntington’s disease); safety and tolerability across agents. Many clinical trials registered but reported limited or no posted results, underscoring the need for transparent outcome reporting.
**• Main outcomes**
- Clioquinol and 5,7-dichloro-2-[(dimethylamino) methyl]-8-hydroxyquinoline showed reproducible biomarker improvements (decreased cerebrospinal fluidand plasma amyloid-beta and cognitive benefits in early-phase Alzheimer’s disease and Huntington’s disease cohorts (Lannfelt et al., 2008; Faux et al., 2010; Huntington Study Group Reach, 2015) (ClinicalTrials.gov ID: NCT00471211, NCT01590888). - Epigallocatechin gallate demonstrated potential to slow cognitive decline and improve brain connectivity in Alzheimer’s disease in a recent trial, supporting neuromodulatory effects tied to metal handling and antioxidant actions (ClinicalTrials.gov ID: NCT03978052). - The αlipoic acid combined with ω3 fatty acids suggested improvements in inflammation, lipid dysregulation, insulin resistance, and cognitive trajectories in Alzheimer’s disease, consistent with a multimodal metabolic–redox mechanism (ClinicalTrials.gov IDs: NCT01058941, NCT00090402). - Several curcumin and epigallocatechin gallate studies listed with no posted results highlight publication and registration gaps that limit definitive conclusions.
**• Safety and feasibility**
- Interventions were generally well tolerated in earlyphase testing, supporting feasibility for longer and larger confirmatory studies.
**• Implications for practice and research**
- Emerging evidence supports the integration of copper homeostasis strategies with standard care, as well as with combinatorial metabolic and antioxidative approaches. - Clinical trial designs should integrate target engagement metrics, such as levels of amyloid-beta molecules in cerebrospinal fluid and plasma, imaging connectivity, and metal concentration measurements, alongside patient-relevant outcomes to ensure both mechanistic validity and clinical relevance.

As mentioned above, dysregulation of cerebral Cu homeostasis is a significant factor in inducing neurotoxicity and AD. Recently, a study using an animal model implicated that chronic Cu accumulation leads to increased oxidative stress in the hippocampus and a decline in learning and memory abilities (Lamtai et al., 2020). In 2022, a clinical study systematically illustrated the complex relationship between cuproptosis and AD (Lai et al., 2022). Using the weighted correlation network analysis (WGCNA) algorithm and machine learning tools, clusters of genes related to cuproptosis were identified. These gene clusters were found to be highly associated with levels of amyloid-beta (Aβ_42_) and β-secretase activity (Lai et al., 2022). In another bioinformatics-based study, seven AD-related cuproptosis genes were identified from the Gene Expression Omnibus, including *IFI30*, *CLIC1*, *LYZ*, *PYGL*, *PLA1A*, *ALOX5AP*, and *A4GAL* (Zhang et al., 2022a). The Gene Ontology (GO) analyses demonstrated that these seven genes were involved in phosphoribosyl pyrophosphate, lipid, and glucose metabolism. Thus, cuproptosis participates in fundamental metabolic processes and is involved in neuronal damage. Experiments conducted on transgenic mouse models and cell lines have shown that the exacerbation of Aβ oligomerization depends on the redox interactions of Cu^2+^/Aβ/H_2_O_2_, which are critical for Aβ toxicity and lead to oxidative stress (Fica-Contreras et al., 2017).

Furthermore, three CRGs and four apoptosis-related genes were identified in patients with AD by Ma et al. (2024), based on the analysis of AD-related microarray datasets from the GEO database. Among these genes, three CRGs—*MAP2K1*, *SLC31A1*, and *PDHB*—were emphasized as potential biomarkers for the diagnosis and treatment of AD. However, the limited sample size of patients and the need for confirmatory experiments, such as cell and animal studies, restrict a comprehensive overview of CRGs involved in the pathogenesis of AD. For instance, employing the CRISPR-Cas9 system to knock out these candidate genes could facilitate the evaluation of AD progression in murine models. The CRISPR-Cas9 genome editing technology may represent a promising therapeutic strategy for the treatment of AD (Khan et al., 2025). The therapeutic potential of the CRISPR-Cas9-based method for managing AD is discussed in Section “Application of CRISPR-Cas9-based gene-editing technology in the management of neurodegenerative diseases “of this review. In another relevant study, Chen et al. (2024) demonstrated that the key cuproptosis gene *FDX1* had a higher expression level in the peripheral blood samples of AD patients and SH-SY5Y cell models compared with controls. The expression level of *FDX1* positively correlated with the *APOE ε4/ε4* genotype of AD patients, and knockdown of this CRG significantly alleviated ROS generation and reduced cuproptosis. This project determined the correlation between cuproptosis and the pathogenesis of AD, suggesting that the expression level of *FDX1* may act as a potential target for the diagnosis and treatment of AD.

### Parkinson’s disease

PD is another critical NDD, characterized by symptoms such as resting tremor, muscular rigidity, postural instability, and bradykinesia. Three hallmarks of PD include the loss of dopaminergic neurons in the substantia nigra, intracellular protein aggregation, and the generation of ROS (Morris et al., 2024). At the molecular pathogenic level, the presence of Lewy bodies—abnormal protein aggregates primarily composed of α-synuclein fibrils—serves as a diagnostic biomarker and plays a key role in the pathogenesis of PD (Morris et al., 2024).

Although α-synuclein fibrils have been determined to play critical roles in the progression of PD, their molecular pathogenic roles are complex and involve several mechanisms. Under normal circumstances, α-synuclein is a soluble presynaptic neuronal protein that regulates synaptic vesicle trafficking and neurotransmitter release. However, severe impairment conditions, such as genetic mutations, can cause α-synuclein to misfold and form insoluble fibrils that exhibit neurotoxicity. These fibrils disrupt synaptic vesicle recycling and lead to the degeneration of dopaminergic neurons. Moreover, the insoluble and aggregated misfolded α-synuclein fibrils impair mitochondrial functions, disrupt protein degradation pathways, induce oxidative stress in neurons, and trigger inflammatory responses (Park et al., 2025). Another pathogenic process involved in PD is the impaired ubiquitin-proteasome pathway (Davidson and Pickering, 2023). The aggregated α-synuclein fibrils may overwhelm the cellular machinery responsible for clearing misfolded proteins, leading to the accumulation of toxic aggregates and further contributing to neuronal damage.

A comprehensive examination of the genotypic characteristics of patients with PD has led to the identification of numerous pathogenic genes (Westenberger et al., 2025). The α-synuclein protein is encoded by the *SNCA* gene, and single-nucleotide polymorphisms (SNPs) in SNCA, as well as gene duplications, contribute to the abnormal folding and aggregation of this protein into oligomers and insoluble fibrils. The identified SNPs in the *SNCA* gene include *A53E*, *A53T*, *A30P*, *H50Q*, *E46K*, and *G51D*. These mutations result in alterations to the solubility of α-synuclein proteins, leading to abnormal aggregation (Mohapatra et al., 2023). *LRRK2* (Leucine-rich repeat kinase 2) gene encodes a protein that exhibits both kinase and GTPase activities. This protein is involved in the regulation of autophagy, mitochondrial functions, chronic inflammation—particularly within the central nervous system—and vesicle trafficking. Notably, the G2019S mutation in *LRRK2* is identified as a risk factor for both sporadic and inherited forms of PD. An increasing body of research indicates that neuroinflammation, driven by glial hyperactivation, significantly contributes to the degeneration of dopaminergic neurons and the pathogenesis of PD (Karayel et al., 2022).

In addition to mutations in autosomal dominant genes, three mutations in autosomal recessive genes (e.g., *PRKN*, *PINK1*, and *PARK7*) also contribute to the genetic etiology of PD (Westenberger et al., 2025). *PRKN*-encoded parkin proteins play a crucial role in degrading misfolded, damaged, or excess proteins by tagging them with ubiquitin molecules. Mutations in *PRKN* are associated with early-onset PD, and the incidence of PD attributed to PRKN variants is similar to that of mutations in the *LRRK2* gene. A relevant epidemiological study found that PRKN mutations account for approximately 1% of PD cases (Westenberger et al., 2024). Loss-of-function and missense mutations are predominant among *PRKN* variants, while copy number variants, particularly deletions of exons, occur more frequently than SNPs (Westenberger et al., 2024). Recent advancements in long-read sequencing techniques have also led to the identification of large DNA region inversions in early-onset PD (Daida et al., 2023). Another pathogenic gene related to PD is *PINK*, which encodes protein phosphatase and tensin homolog-induced kinase 1. This kinase participates in mitochondrial quality control by translocating to the outer mitochondrial membrane, where it identifies misfolded proteins within the mitochondrial matrix. Under conditions of mitochondrial stress, these kinases adopt a stabilized conformation and accumulate on dysfunctional mitochondria to facilitate the phosphorylation of parkin proteins, thereby promoting mitophagy (Narendra and Youle, 2024). Missense variants are the predominant mutations in this gene, while structural variants account for only a small percentage (Westenberger et al., 2025). *PARK7* encodes the deglycase DJ-1, which functions as an oxidative stress sensor and antioxidant. It plays a critical role in protecting against neurodegeneration by acting as a protease and molecular chaperone (Lv et al., 2025). However, mutations in the *PARK7* gene that lead to PD are relatively rare, with the majority being missense mutations and loss-of-function variants (Westenberger et al., 2025). In addition to inheritable factors, most cases of PD are related to aging and environmental factors (Perinan et al., 2022). Although the exact pathogenesis of PD has not been fully elucidated, several studies have demonstrated that oxidative stress and mitochondrial dysfunction are contributing factors to the progression of PD (Liu et al., 2025a).

As a critical cofactor for numerous enzymes, Cu ions are significant for biochemical functioning. However, excess free Cu ions can bind to cysteine residues of proteins, resulting in the inactivation of their enzymatic activity (Letelier et al., 2009). In this regard, the administration of Cu ions has been shown to significantly reduce the binding of D2 dopamine receptors with [^3^H]-spiperone due to the ability of Cu ions to alter the binding sites of dopamine receptors (Scheuhammer and Cherian, 1985). Additionally, Cu ions participate in the conversion of the superoxide anion and hydrogen peroxide into the hydroxyl radical intermediate, leading to an increase in the level of ROS (Jomova et al., 2025; **Figure 5**). Further evidence elucidating the pathogenic role of Cu in PD indicates that it enhances the aggregation of misfolded α-synuclein. Under normal circumstances, soluble and unfolded α-synucleins predominantly adopt an α-helical conformation. However, in the progression of PD, these unfolded proteins aggregate into oligomers and fibrils that are rich in β-sheet structures (Abrao-Nemeir et al., 2025). In this process, Cu ions are determined to promote the formation of β-sheet structures, thereby enhancing the aggregation of α-synuclein (Scolari Grotto and Glaser, 2024). An *in vivo* study has shown that Cu ions are present at synapses, promoting α-synuclein aggregation (Scolari Grotto and Glaser, 2024). The role of Cu ions in enhancing the progression of PD is illustrated in **Figure 5**.

During the progression of PD, Cu binds to lipoylated E2 subunits within pyruvate dehydrogenase and α-ketoglutarate dehydrogenase—mitochondrial dehydrogenase complexes—causing protein aggregation that impairs TCA cycle flux and NADH production. These effects lead to mitochondrial dysfunction and deficits in bioenergy production in the dopaminergic neurons of the substantia nigra. Furthermore, in animal models and postmortem PD tissues, cuproptosis induces destabilization of Fe-S cluster proteins, compromises the electron transport chain, and disrupts redox homeostasis (Huang et al., 2024). Several CRGs, such as *NFE2L2*, *MTF1*, and *ATP7B*, have been identified as associated with the pathogenesis of PD, offering a potential new avenue for treatment. In another study from China, the application of artesunate was utilized to target the 6-hydroxydopamine-induced dopamine neurotoxicity in astrocytes. Interestingly, the presence of artesunate reduced intracellular levels of Cu^2+^ and rebalanced the expression of several proteins involved in the cuproptosis pathways, such as FDX1, CTR1, and DLAT. This study indirectly highlighted the pathogenic roles of cuproptosis in PD (Cheng et al., 2025).

### Huntington’s disease

HD is a hereditary neurodegenerative disorder characterized by the progressive degeneration of neurons in specific regions of the brain. It is caused by a mutation in the huntingtin (*HTT*) gene located on human chromosome 4, which leads to the production of an abnormal form of the huntingtin protein (Jiang et al., 2023). The pathogenesis of HD involves several key mechanisms: (1) aggregation of the mutant huntingtin protein, (2) disruption of cellular functions, (3) excitotoxicity, (4) oxidative stress, (5) impaired energy metabolism, and (6) neuroinflammation. Briefly, the mutant huntingtin protein contains an expanded polyglutamine repeat (CAG codon repeats), which makes it prone to misfolding and aggregation. This aggregation subsequently impairs protein degradation pathways, leads to mitochondrial dysfunction, alters gene transcription, and disrupts intracellular signaling (Heinzmann et al., 2023).

In patients with HD, the mutant *HTT* disrupts glutamate receptors, resulting in excessive Ca^2+^ influx into neurons, which causes neuronal damage and degeneration (Guo and Ma, 2021). Another pathogenic hallmark of HD is the accumulation of oxidative stress-induced neuronal damage, which occurs due to an imbalance between ROS generation and detoxification pathways. Additionally, mitochondrial dysfunction impairs energy metabolism and disrupts glucose metabolism, further contributing to neuronal dysfunction.

The rise of neuroinflammation is another hallmark in patients with HD, resulting from the activation of microglia and astrocytes in response to the presence of mutant HTT aggregates. The presence of reactive astrocytes in presymptomatic patients with HD, along with the active status of these cells, may serve as a potential biomarker for evaluating disease severity. Additionally, a significant accumulation of reactive microglia has been observed in the striatum and cortex of patients with HD (Saba et al., 2022). Furthermore, microglial cells release pro-inflammatory molecules, leading to chronic neuroinflammation, which contributes to the detrimental effects seen in typical motor, cognitive, and psychiatric symptoms (Saba et al., 2022).

The exact role of Cu in HD remains unclear, although the basal ganglia are known to be sensitive to elevated levels of Cu. For instance, 40% of patients with Wilson’s disease, which is characterized by increased Cu levels in the liver, exhibit Cu-induced striatal degeneration (Tinaz et al., 2021). Emerging evidence indicates increased levels of Cu in cerebrospinal fluid (CSF) samples from both early and late-stage HD patients, as well as elevated Cu content found in the striatum of post-mortem HD patients (Pfalzer et al., 2022). Additionally, accumulated Cu ions in the brains of individuals with HD have been found to interact chemically with both normal and mutated huntingtin proteins. Cu dysregulation may be involved in the progression of HD by contributing to oxidative stress and neuronal dysfunction. Unlike related studies on AD and PD, the specific roles of cuproptosis in the pathogenesis of HD are not as well established, and the association between cuproptosis and the pathogenesis of HD requires further elucidation.

### Amyotrophic lateral sclerosis

ALS is a NDD characterized by the progressive loss of motor neurons in the brain and spinal cord. This disease results in paralysis and death within 2–5 years. The exact pathophysiological mechanisms involved in ALS are still undetermined, and effective treatments for this disease remain obscure. Reasons for selective loss of motor neurons have been determined with oxidative stress, mitochondrial dysfunction, glutamate excitotoxicity, and neuroinflammation (Liu et al., 2023).

Genetically related reasons for ALS pathogenesis include the mutations in *C9orf72* and *SOD1*. The causative mutation of *C9orf72* is an abnormal hexanucleotide (GGGGCC, G4C2) repeat expansion located in the first intron of *C9orf72*, while *SOD1* mutations are mainly SNP variants (Mizielinska et al., 2025). Functions of the *C9orf72*-encoded protein are significant to maintain the normal cellular functions and pathways that involve the regulation of autophagy–lysosome pathway, synaptic function and axonal maintenance, nuclear-cytoplasmic transport, and immune regulation (Pang and Hu, 2021; Smeyers et al., 2021). The *C9orf72*-encoded protein is part of a complex with SMCR8 and WDR41, acting as a GDP-GTP exchange factor for RabGTPases. C9orf72-SMCR8-WDR41 complex associates with the FIP200/Ulk1 complex that is essential for autophagy initiation. The *C9orf72* knockout mice with increased levels of autophagy and lysosomal proteins, and the autophagy pathways are compromised in both liver and spleen (Sullivan et al., 2016). Furthermore, C9orf72 plays a crucial role in axon growth and trafficking. The overexpression of this gene results in the enhancement of motor neuron axon growth, whereas a reduction in its expression leads to adverse effects. This phenomenon may be attributed to the interaction between C9orf72 and cofilin, which regulates actin dynamics (Shehjar et al., 2024). *SOD1* encodes an antioxidant enzyme that facilitates the conversion of ROS into less harmful molecules. In contrast to *C9orf72*, *SOD1*-related ALS is characterized by a toxic gain-of-function mutation rather than a loss-of-function mutation. Nearly 200 variants of *SOD1* have been identified in ALS cases. The mutation causing dysregulation or dysfunction of SOD1 can lead to an imbalance between the production of ROS and antioxidant defense, resulting in oxidative stress. The three most commonly identified mutations in the *SOD1* gene are D90A, A4V and G93A. For example, the *SOD1*^G93A^ transgenic mouse model has been developed to study ALS, as it effectively mimics the symptoms of the disease (Salvany et al., 2022). Mutant *SOD1* proteins exhibit a propensity to mis-folding, aggregate, and form intracellular inclusions within motor neurons, thereby disrupting normal cellular processes. The localization of misfolded SOD1 to the mitochondria of motor neurons leads to mitochondrial swelling and dysfunction, which subsequently impairs neuronal metabolism and survival (Salvany et al., 2022; Guo et al., 2024).

**Figure 6:**
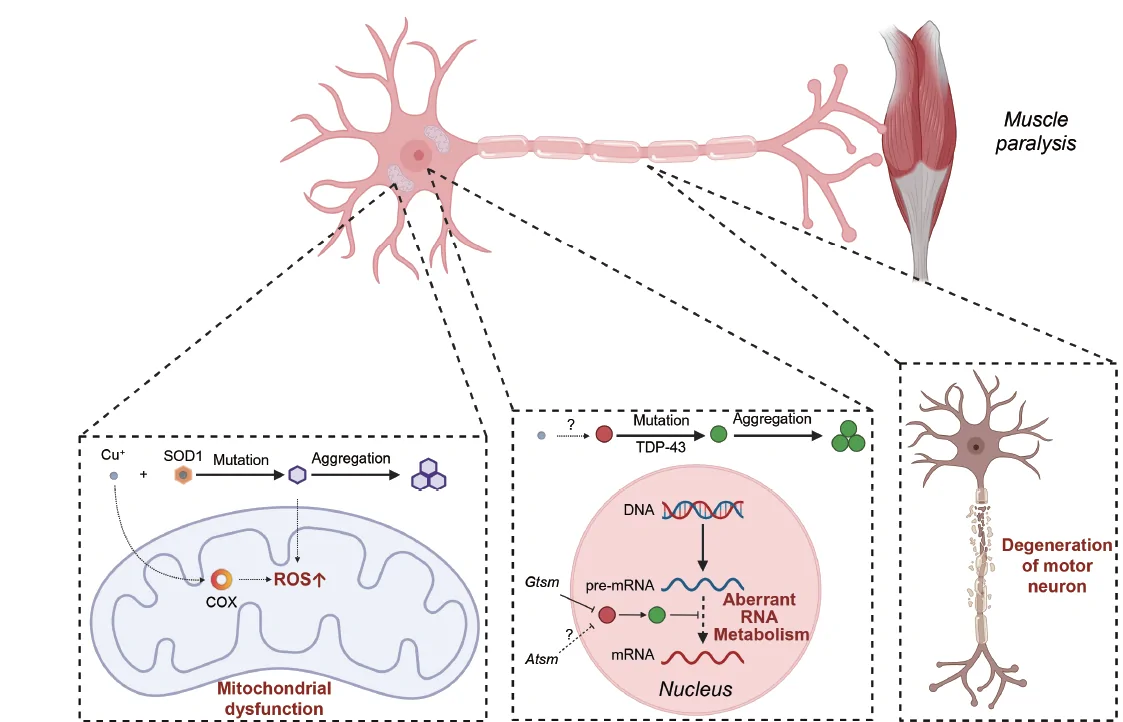
Schematic model of Cu in the pathogenesis of ALS. Three Cu-related pathways are involved in the pathogenesis of ALS, which are SOD1 mutation, mitochondrial dysfunction, and the TDP-43 protein. SOD1 is the Cu binding protein and localizes in the cytoplasm, nucleus, and intermembrane space of mitochondria. The functions of SOD1 involve the antioxidant defense. Mutated SOD1 is disabled to bind to the Cu ions and loses of function in oxidant detoxification. Cu also acts as an essential cofactor for COX, which is a key enzyme in the electron transport chain. Dysregulation of Cu leads to the impaired functions of COX. TDP-43 protein mutation is another key issue in leading the ALS via impaired RNA metabolism, though the exact pathogenic role of Cu is uncertain. However, Cu chelators displayed neuroprotective effects through the blockage of the formation of TDP-43. Additionally, the Cu dyshomeostasis induced increased ROS, mitochondrial dysfunction, and aberrant RNA metabolism that finally led to the degeneration of motor neurons and enhanced the pathogenic progression of ALS. Created in BioRender. Du, W. (2025) https://BioRender.com/q0a16oz. ALS: Amyotrophic lateral sclerosis; atsm: diacetylbis (-methylthiosemicarbazonato) Cu (II) (Cu (II)); COX: cytochrome c oxidase; Cu: copper; gtsm: glyoxalbis (-methylthiosemicarbazonato) Cu (II) (Cu (II)); ROS: reactive oxygen species; SOD: superoxide dismutase; TDP-43: TAR DNA-binding protein 43.

Recently, emerging evidence suggests that Cu regulation and cuproptosis may play a role in the pathogenesis of the disease. Clinical studies have reported that ALS patients with abnormal Cu metabolism have elevated Cu levels in both CSF and serum (Kamalian et al., 2023; Gao et al., 2025). In this section, we highlight three Cu-related pathways involved in the pathogenesis of ALS: SOD1 mutation, mitochondrial dysfunction, and TDP-43 (**Figure 6**). As mentioned above, SOD is an essential enzyme for maintaining cellular redox balance and protecting cells from oxidative stress. Based on their metal ion binding and localization, SODs are classified into three main forms, with SOD1 being specifically related to the pathogenesis of ALS. SOD1, also referred to as Cu/Zn SOD, is located in the cytoplasm, nucleus, and intermembrane space of mitochondria. SOD2 and SOD3 are known as manganese SOD and extracellular SOD, respectively. Thus, Cu and Zn ions are required for the deactivation of toxic superoxide radicals. In ALS cases with SOD1 mutations, the mutated SOD1 enzyme exhibits abnormal binding with Cu, leading to protein misfolding, dysfunction, aggregation, and toxicity. Impaired SOD1 functions further induce oxidative stress and neuronal death in ALS patients. In transgenic mice expressing the human mutant *SOD1*^G93A^, intracellular Cu homeostasis is dysregulated, resulting in Cu ion accumulation in the spinal cord; these outcomes significantly contribute to the pathogenesis of ALS (Tokuda et al., 2013). Therefore, it is critical to decipher the relationship between Cu and *SOD1* to develop therapeutic strategies aimed at mitigating the toxic effects of mutant *SOD1* in ALS (Min et al., 2024).

Cu dysregulation and mitochondrial dysfunction are both implicated in the development of ALS (Dafinca et al., 2021). Cu is an essential cofactor for several enzymes involved in mitochondrial function, including COX, which plays a key role in the electron transport chain. COX is responsible for the final step in cellular respiration, where it transfers electrons to oxygen to generate ATP (Maung et al., 2021). Mitochondrial dysfunction has been linked to many aspects of ALS pathogenesis, contributing to the selective vulnerability of motor neurons (Meng et al., 2025). Cu dysregulation can directly impact mitochondrial function; excessive Cu accumulation or deficiency can disrupt COX activity in mitochondrial respiration. This disruption may impair ATP production, increase ROS generation, and contribute to mitochondrial dysfunction in ALS.

Another identified factor in the pathogenesis of ALS is the aggregation of TDP-43 protein (TAR DNA-binding protein 43). This nuclear protein, which is responsible for RNA processing and metabolism—including transcription, splicing, transport, and translocation—becomes abnormally aggregated and mislocalized in the cytoplasm of neurons in patients with ALS. The aggregated TDP-43 is considered one of the pathological hallmarks of ALS and contributes to neuronal dysfunction and degeneration. Although the exact mechanisms underlying TDP-43 aggregation and toxicity are not fully understood, the accumulation of truncated forms of TDP-43 is thought to disrupt normal RNA processing in affected neurons (Liao et al., 2022). Two studies have produced conflicting results regarding the association between copper (Cu) and the pathogenic roles of TDP-43 in ALS. On one hand, Cu chelators, such as glyoxalbis(-methylthiosemicarbazonato) Cu(II) (Cu(II)) and diacetylbis(-methylthiosemicarbazonato) Cu(II) (Cu(II)), displayed neuroprotective effects and blocked the formation of TDP-43 in SH-SY5Y cell lines (Parker et al., 2012). On the other hand, a 2023 study from Australia highlighted the limited effectiveness of Cu(II) in reducing neuronal death or astrogliosis in ALS patients (Yang et al., 2023). These conflicting outcomes reflect the unclear association between Cu and TDP-43 pathogenicity in ALS. Koski et al. (2021)found increased levels of Cu ions in the spinal cords of *TDP-43*^A315T^ transgenic mice, although metal levels in the brain remained unchanged. The overexpression of mutant TDP-43 proteins significantly impaired locomotive activities and disrupted metal ion homeostasis in the spinal cord in this animal model, but cognitive decline was not affected.

Although Cu dysregulation can lead to the accumulation of Cu in mitochondria, there have been limited studies demonstrating the association between cuproptosis and ALS pathogenicity. One recent study utilized the Gene Expression Omnibus (GEO) online database to predict CRGs that may be related to the pathogenesis of ALS (Jia et al., 2024). While the Support Vector Machine (SVM) model exhibited the best discrimination properties in this study, either the prediction model or the selected CRGs need to be further validated through molecular experiments and animal models.

### Prion diseases

**Figure 7:**
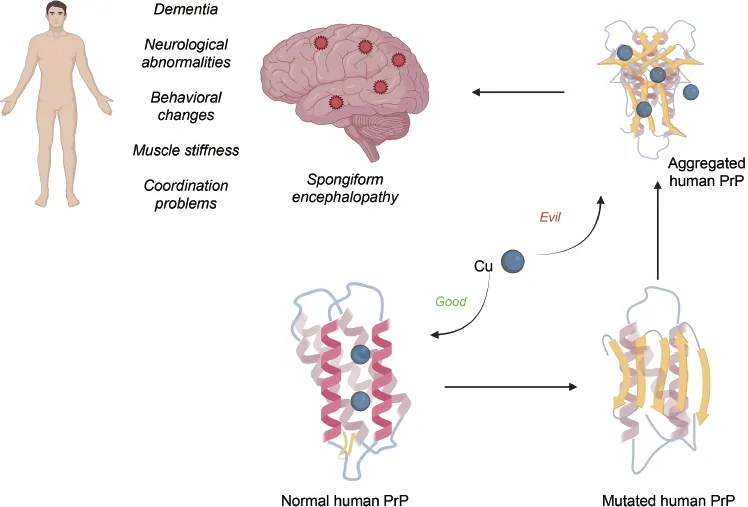
A diagram illustrating the pathogenic roles of Cu in prion disease. The presence of mutated and aggregated prion protein (PrP) in the brain is the underlying cause of prion disease. Cu ions have been found to interact with misfolded PrPs and promote their aggregation and toxicity at the conserved His residues in the N-terminal domain of PrPs. The consequences of aggregated PrPs involve dementia, neurological abnormalities, behavioral changes, muscle stiffness, and coordination problems. Created in BioRender. Du, W. (2025) https://BioRender.com/g7tgmrg.

Prion diseases are a group of rare and fatal neurodegenerative disorders that affect both humans and animals and are also known as transmissible spongiform encephalopathies (Pinar-Morales et al., 2023). Several pathological hallmarks have been identified in these diseases, including extensive spongiform encephalopathy, gliosis, and the presence of aggregated prion proteins. Importantly, aggregated prion proteins exist in various sizes, including small soluble oligomers and long, thin, unbranched fibrils, which vary depending on the specific disease (Sigurdson et al., 2019). There are four primary subtypes of prion diseases, each characterized by distinct clinical features including Creutzfeldt-Jakob disease, variant Creutzfeldt-Jakob disease, Gerstmann-Sträussler-Scheinker syndrome, and fatal familial insomnia (Zerr et al., 2024). Patients with prion diseases typically suffer from rapidly progressive dementia, neurological abnormalities, and behavioral changes. As the severity of the disease increases, patients may experience muscle stiffness, coordination problems, difficulty speaking or swallowing, severe disability, and eventually death (**Figure 7**). The exact pathogenic causes of these diseases are complex and multifactorial, including genetic mutations, sporadic cases with no known cause, and transmission through contaminated tissues or medical procedures. Currently, regardless of the diagnosis or management, prion diseases remain a significant challenge for clinicians (Shim et al., 2022).

These diseases are caused by the conformational conversion of cellular prion protein (PrP^C^) to the aggregated form, PrP^Sc^ (Baiardi et al., 2023). To date, 42 different mutations in the *PRNP* (PrP gene) have been identified, resulting in severe pathogenic consequences. Recombinant human PrP consists of 253 amino acids and contains two signal peptides (1–22 and 231–253). The region from residues 23 to 230 includes two C-terminal fragments (90–230 and 121–230) and one globular domain (125–228). This globular domain comprises three α-helices and a short anti-parallel β-sheet. Additionally, human PrP mutations have been determined in residues 90–230 (Appleby et al., 2022). Cu is essential for maintaining the native and normal form of PrP, and the biological functions of this protein rely on Cu binding (Hara et al., 2023). Cu-binding sites have been identified in multiple histidine (*His*) residues within the N-terminal domain of human PrP, including positions 96 and 111 (Kawahara et al., 2021). However, Cu has also been shown to interact with misfolded PrPs, promoting their aggregation and toxicity. The conserved and Cu-bound *His* residues facilitate the reduction of Cu from Cu^2+^ to Cu^+^, and PrP has been shown to affect brain Cu levels (Hara et al., 2023). In animal models, the depletion of PrPs, such as in *PrP*^0/0^ mice, significantly impairs Cu metabolism and Cu-dependent oxidase activity. It is believed that the presence of Cu in the prion protein alters its conformation and stability, facilitating the formation of PrP aggregates (do Amaral et al., 2023).

Although previous studies have demonstrated that Cu may interact with mutated PrP and enhance aggregation and toxicity, the exact physiological function of Cu ions in PrP is still being investigated, and the beneficial or detrimental roles of Cu in prion diseases remain under debate (do Amaral et al., 2023; Hara et al., 2023). The pathogenic role of Cu in prion diseases may vary depending on the specific type of prion disease and other factors. A search of the PubMed database revealed no related papers describing the pathogenic roles of cuproptosis in prion diseases. Therefore, the association between Cu and the pathogenesis of prion diseases is still an ongoing topic that requires further research.

## Potential Applications of Therapeutic Strategies Targeting Copper Metabolism and Cuproptosis in Neurodegenerative Diseases

### Targeting cuproptosis: Therapeutic targets for neurodegenerative diseases

As previously discussed, the dysregulation of Cu metabolism and the process of cuproptosis are critical factors in the pathogenesis of NDDs. The identification of cuproptosis as a significant therapeutic target has garnered attention, and the exploration of CRGs may provide a promising strategy for managing NDDs. Utilizing bioinformatics methodologies and systematic analyses of clinical data, numerous genes, gene clusters, and molecular pathways have been identified that elucidate the causal relationship between cuproptosis and NDDs. For instance, *IFI30*, *CLIC1*, *LYZ*, *PYGL*, *PLA1A*, *ALOX5AP*, and *A4GAL* have been implicated in cuproptosis related to AD (Zhang et al., 2022a). Additionally, other CRGs such as *MAP2K1*, *SLC31A1*, and *PDHB* have been identified as potential biomarkers for the diagnosis and treatment of AD (Ma et al., 2024).

Based on the analyses of the GSE63060 and GSE63061 datasets, which include RNA-seq data from blood samples of 104 healthy subjects and 145 patients with AD, 12 CRGs were identified (Nie et al., 2023). These genes consist of seven pro-cuproptosis genes: *PDHA1*, *DLAT*, *LIAS*, *FDX1*, *PDHB*, *LIPT1*, and *DLD*; three anti-cuproptosis genes: *GLS*, *MTF1*, and *CDKN2A*; as well as two copper transporter genes: *ATP7B* and *SLC31A1*. Additionally, the differentially expressed CRGs were used to categorize the 145 AD patients into two subgroups, a classification that could be used to develop diagnostic models and facilitate the analysis of immune infiltration during AD progression. In another bioinformatics study, Hu et al. (2024) identified five critical CRGs associated with AD by screening differentially expressed genes in 356 AD patients and 189 healthy subjects. These identified CRGs were subsequently validated in AD murine models and SH-SY5Y cell lines, revealing four downregulated genes (*DLD*, *FDX1*, *GLS*, and *PDHB*) and one upregulated gene in AD patients compared with healthy controls. The distinct expression patterns of these target genes may provide a novel perspective for the diagnosis and prognosis of AD (Hu et al., 2024).

Although cuproptosis is associated with the progression of PD, the number of related studies remains limited (Liu et al., 2025a; Wang et al., 2025a). Six CRGs have been identified in the pathogenesis of PD, including *KIAA0319*, *AGTR1*, *NFE2L2*, *SLC18A2*, *MTF1*, and *ATP7B*. These findings provide a novel direction for the treatment of PD (Wu et al., 2023; Zhang et al., 2023). Moreover, numerous studies have focused on elucidating the relationship between immune response or immune infiltration and CRGs in the progression of PD (Zhang et al., 2023, 2024a; Zhong et al., 2023). Increased expression levels of three CRGs—*SLC18A2*, *SLC6A3*, and *SV2C*—were observed in PD patients, and the upregulation of these genes also correlates with the dysregulation of host immune responses. Thus, CRGs that enhance PD progression may negatively impact the host immune system (Zhang et al., 2024a).

Wu et al. (2023) identified three CRGs in PD patients, including two upregulated genes, *MTF1* and *NFE2L2*, alongside a downregulated gene, *ATP7B*. Using a machine learning model and a drug prediction model (The Drug Gene Interaction Database, DGIdb), they determined that *MTF1*, which encodes pyrithione zinc, may serve as a potential target for PD, while bardoxolone methyl, dimethyl fumarate, and sulforaphane were selected for their potential to target the upregulation of *NFE2L2*. Copper chelators, such as penicillamine and trientine, may act as potential therapeutic agents to counteract cuproptosis-related PD progression (Wu et al., 2023). This study provides a promising research model for understanding cuproptosis-induced PD and for identifying potential anti-PD therapies; however, the efficacy and safety of these proposed treatments require further evaluation in both animal models and human clinical trials.

### Copper chelators for the treatment of neurodegenerative diseases

Cu dyshomeostasis is a critical issue for inducing NDDs. Thus, targeting Cu dysregulation is an increasing topic for treating NDDs, though Cu-related therapies are still in the early stages of research development. Up to now, several approaches have been explored to target Cu dysregulation and mitigate its detrimental effects.

Cu chelation therapy involves the use of specific compounds that bind to Cu ions and facilitate their removal from the body. This therapeutic strategy relies on the application of metal-protein attenuating compounds, which are non-toxic and highly efficient in interacting with metal ions. This interaction is critical for maintaining the homeostasis of intracellular metal ions (Fasae et al., 2021). This type of agent includes several chelators, such as clioquinol (CQ) and 5,7-dichloro-2-[(dimethylamino) methyl]-8-hydroxyquinoline (PBT2). The chemical principle of chelation involves binding ions, which leads to the formation of ringed-structural molecules through the formation of covalent and coordinate linkages (Chen et al., 2023). Previous studies from different groups have elucidated that chelator display neuroprotective activity against metal-mediated NDDs (Chen et al., 2023, 2025; **Additional Table 1**).

The chelating preference of CQ is for Cu and Zn. As early as 2001, CQ was reported to dissolve Aβ deposits, with this inhibition demonstrated in mouse models (Cherny et al., 2001). The application of this chelator has resulted in a 50% reduction in brain Aβ levels and has mitigated Aβ toxicity. Furthermore, in the APP/PS1 transgenic mouse model, CQ inhibited Aβ generation and suppressed the pathogenesis of AD (Wang et al., 2012). In AD patients, CQ has been tested in several Phase II trials, showing its ability to reduce Aβ aggregation and improve cognitive function (Jenagaratnam and McShane, 2006; Bush, 2008). A study investigating the therapeutic applications of astragalin in the context of AD demonstrated that this natural flavonoid has beneficial effects on improving learning and cognitive abilities in mice following hippocampal injections of Aβ_1–42_ and Aβ_25–35_. However, the presence of CQ was found to inhibit these beneficial effects of astragalin, indicating that the therapeutic applications of CQ warrant further investigation (Wang et al., 2025b). In HD, CQ has been shown to reduce the expression of *HTT* and alleviate pathology in mouse models (Nguyen et al., 2005). Recent findings indicate that CQ has demonstrated the ability to prolong lifespan and enhance motor activity in an AD *Drosophila melanogaster* model (Liu et al., 2025d).

PBT2 is an orally active Cu/Zn ionophore that has shown positive effects in restoring cognition in AD mouse models. This Cu-based chelator underwent a Phase II trial and also demonstrated improvement in cognitive performance in patients with AD (Faux et al., 2010). In another Phase II clinical trial, PBT2 dramatically reduced the concentration of Aβ_1–42_ in the CSF samples of patients with AD; however, this chelator did not impact serum Cu^2+^ concentrations (Lannfelt et al., 2008). In addition to its therapeutic potential in AD, the safety and efficacy of PBT2 have been tested in patients with HD. In a randomized, double-blind, placebo-controlled trial, PBT2 (250 mg/d) improved cognitive outcomes; however, it is important to be aware of the dosage and potential side effects (Huntington Study Group Reach, 2015).

Curcumin is another widely used chelator of Cu, Fe, and Zn and exhibits inhibitory effects on Aβ aggregation. In an AD mouse model, curcumin enhances the upregulation of the Wnt/β-Catenin pathway and brain-derived neurotrophic factor, thereby improving neurogenesis. The administration of 100 mg/kg curcumin alleviates impaired neurogenesis and improves cognitive function in Aβ_1–42_ mouse models (Lou et al., 2024). In another 5×FAD mouse model, curcumin reduces Aβ aggregation and mitigates memory decline by inhibiting the expression of β-site amyloid precursor protein cleaving enzyme 1 (BACE1) (Zheng et al., 2017). *In vitro* experiments have shown that curcumin enhances microglial phagocytosis and clearance of Aβ; it also reduces Aβ accumulation in neuronal cell lines (Cole et al., 2007). In the neurotoxicity induced by 6-hydroxydopamine in SH-SY5Y and MES23.5 cell lines, curcumin mitigates detrimental effects by enhancing mitochondrial protection, attenuating apoptosis, and inhibiting the generation of ROS (Wang et al., 2009; Jaisin et al., 2011). Furthermore, the interaction and modulation of curcumin with molecular targets, such as the antioxidant system, inflammatory cytokines, and transcription factors, contribute to its neuroprotective effects. This chelator has demonstrated beneficial effects in various types of neural cells, including neurons (Ding et al., 2025b), astrocytes (Maurya et al., 2021), and microglia (Xu et al., 2025b). Another mechanism by which curcumin inhibits neuroinflammation was demonstrated in an apolipoprotein E4 (*APOEε4*) transgenic mouse model, where it suppresses the endoplasmic reticulum pathway (Kou et al., 2021). In addition to its role as a Cu chelator, curcumin acts as an antioxidant and anti-inflammatory agent, influencing the activation of the NF-κB signaling pathway in AD and PD, resulting in the downregulation of cytokine expression (Esmaealzadeh et al., 2024).

From tea, epigallocatechin-3-gallate (EGCG) has been identified as the active agent with high bioavailability. This chemical compound exhibits a chelating preference for Cu, Fe, and Zn (Lin et al., 2022). There is evidence that EGCG binds directly to the β-sheet of Aβ, remodeling the Aβ conformation without the toxic oligomeric intermediates, thereby significantly decreasing the toxicity associated with Aβ aggregation (Li et al., 2024). In primary microglia, EGCG has been found to reduce microglial inflammation by inhibiting the activation of NLRP3 inflammasomes and caspase-11-dependent inflammasomes via the TLR4/NF-κB pathway in an APP/PS1 transgenic mouse model (Zhong et al., 2019). Other beneficial effects of EGCG in treating AD include the inhibition of mitochondrial dysfunction and ROS generation, demonstrating great therapeutic potential in neuroprotection (Payne et al., 2022).

LA acts as a chelator for Cu and possesses anti-inflammatory and antioxidant properties. This chelator has been implicated in cognitive and memory improvement by reducing reactive astrocyte proliferation in a neurodegenerative mouse model (Memudu and Adewumi, 2021). In a PD mouse model, LA significantly attenuated neuroinflammation by inhibiting the expression of NF-κB, TNF-α, and inducible nitric oxide synthase (Zhang et al., 2022b). In AD neuron-based experiments, LA functions as a chelator to maintain normal Cu levels (Metsla et al., 2022). To enhance selectivity for chelating Cu ions, a chromone-LA hybrid was designed through a click reaction. This fabrication resulted in significant inhibition of ROS and moderate inhibition of Aβ aggregation (Jalili-Baleh et al., 2021).

As Cu is involved in numerous biological processes in the human body, the physiological toxicity of chelator-dependent therapy is a concern. The development of a cerebral drug delivery system could be an ideal option for enhancing Cu-chelator-based therapies for NDDs.

### Targeting Cu: Therapeutic applications of nanoparticles in neurodegenerative diseases

In previous sections, we have emphasized the pathogenic roles of Cu dyshomeostasis in NDDs. In this subsection, we discuss the therapeutic applications of nanoparticles in the treatment of NDDs. Despite a relatively comprehensive understanding of the pathogenesis of AD, treatment options remain limited due to various challenges. Among these obstacles, the phospholipid bilayer-based BBB constitutes a significant impediment, restricting the entry of conventional drugs and leading to suboptimal efficacy of anti-AD therapies. Nanosized drug carriers, however, hold great potential for traversing the BBB (Passeri et al., 2022; Pawar et al., 2025).

Nanoparticles (NPs, with a particle size of less than 400 Da), nano-sized liposomes, and exosomes have demonstrated significant capabilities in crossing the BBB (Sun et al., 2023). Selenium-doped carbon dot-based nanoparticles have been synthesized and evaluated for their ability to inhibit Aβ aggregation, scavenge ROS, and chelate Cu. In this NP system, selenium and glutathione precursors were carried by the carbon dot-based nanoparticles across the BBB and released within the brain for ROS elimination and Cu chelation. This NP system demonstrated relative effectiveness in alleviating the progression of AD (Shao et al., 2025). In another independent study, a research team from China synthesized porphyrin-substituted phenylalanine-phenylalanine nanoparticles and demonstrated that these NPs significantly inhibit Aβ aggregation and the Cu-induced generation of ROS. Moreover, these NPs enable the transport of fluorescent probes across the BBB for the detection of Aβ oligomers. This nanoparticle system exhibits considerable potential for both the treatment and diagnosis of AD (Xia et al., 2025). The BACE1 is responsible for Aβ synthesis and cleavage, making it a critical target for AD therapy (Bi et al., 2025). Another study formulated curcumin molecules into carrier-free curcumin NPs, which were administered intranasally to AD transgenic mice. This treatment inhibited Aβ aggregation and enhanced Aβ clearance by microglia. Additionally, it promoted microglial polarization from the M1 to the M2 phenotype by inhibiting the TLR4/NF-κB pathway, thereby reducing neuroinflammation induced by M1-like microglia (Feng et al., 2024).

By integrating BACE1-siRNA, metallothionein (cysteine-rich proteins that bind Cu for cellular Cu homeostasis and excessive Cu detoxification), and ruthenium (Ru) complexes into Prussian blue nanoparticles (designated as PRM-siRNA). Under the near-infrared light irradiation, this nanosystem enables to traverse the BBB to inhibit Aβ production and enhance Cu chelation through the actions of BACE1-siRNA and metallothionein, respectively. The roles of Ru in this system include monitoring and tracking the degradation and aggregation of Aβ. The therapeutic potential of this NP has been validated in APP/PS1 mice, where administration of PRM-siRNA significantly improved learning and memory, activated astrocytes and microglial cells, and reduced neuronal loss in this AD mouse model (Ding et al., 2025a).

### Application of CRISPR-Cas9-based gene-editing technology in the management of neurodegenerative diseases

CRISPR-Cas9 based gene-editing technology represents a novel therapeutic strategy for the management of NDDs. This system was firstly identified in bacteria and archaea in 2007 and was initially recognized as a prokaryotic defense mechanism for the elimination of invading DNA molecules (Barrangou et al., 2007). This method currently represents a promising approach for the management of AD via correcting the mutation of *PSEN1* and *APP* genes (Bhardwaj et al., 2022; Thapar et al., 2024). PS1 acts as a central pathogenic factor in the early-onset AD by participating the generation of Aβ peptides. In 2016, two studies utilized CRISPR-Cas9 system to correct the amino acid substitutions in *PSEN1* gene (*A79V* and *L150P* variants) in AD patients derived human induced pluripotent stem cells (Pires et al., 2016; Poon et al., 2016). Another amino acid substitution (M146L) in PS1 amino acid sequence results in the increased production of Aβ_42_ peptides, which exhibit a greater capability for aggregation. In human fibroblast cell lines, the CRISPR-Cas9 system disrupts this variant allele, leading to a significant reduction in the Aβ_42_/Aβ_40_ ratio. CRISPR-Cas9 displayed therapeutic potential for early-onset AD with M146L mutation in PS1 (Konstantinidis et al., 2022). Another target of CRISPR-Cas9 in the management of AD is the mutated APP gene. The *APP Swedish* (*APP*^sw^) mutation results in increased β-secretase cleavage of Aβ and serves as a dominant factor in patients with inherited forms of AD. Additionally, the CRISPR-Cas9 method is not only a therapeutic strategy but also serves as a useful tool for identifying therapeutic targets for AD. A previous study has demonstrated that cAMP response element-binding protein-regulated transcription coactivator 1 (CRTC1) is essential for synaptic plasticity, cognitive, memory, learning, and regulation of neuronal functions-associated gene expression. The mutation of this gene is implicated in the pathogenesis of AD (Zhang et al., 2025). This study has demonstrated that the diminished S-nitrosylation of CRTC1 can be achieved using CRISPR-Cas9 to mutate the cysteine at the 216 position of the amino acid to alanine in hiPSC-derived cerebrocortical neurons harboring a single allele of the *APP*^SW^ mutation, resulting in rescued synaptic plasticity in a 5×FAD mouse model. CRISPR-Cas9 has revealed the pathogenic roles of S-nitrosylation in CRTC1 and has provided a promising target for AD (Zhang et al., 2025). In addition to the deletion or correction of pathogenic SNPs in target genes associated with AD, CRISPR-Cas9 also facilitates the introduction of SNPs to potentially manage the condition. Research conducted on healthy and aged populations in Iceland identified the beneficial A673T mutation in the *APP* gene, which results in a reduction of β-secretase cleavage. CRISPR-Cas9 was used to modify the APP protein by converting the alanine codon to a threonine codon in HEK293T cells and SH-SY5Y neuroblastoma cells. Consequently, the introduction of the A673T mutation led to a significant reduction of 53% in Aβ peptide accumulation in HEK293T cell lines. Although this study was conducted under *in vitro* conditions, it demonstrates the considerable potential of genome editing as a therapeutic approach for AD (Guyon et al., 2021).

In addition to its therapeutic applications, the CRISPR-Cas9 system demonstrates considerable potential in elucidating novel pathogenesis associated with AD. Late-onset AD is a severe form of the disease, and several gene variants have been identified that are linked to this subtype. One study used the CRISPR-Cas9 technology to introduce a rare functional variant of the *SHARPIN* gene, specifically rs572750141 (G186R), into *HEK293* cells. The homozygous knock-in cells harboring this variant exhibited a significant increase in Aβ_40_ and Aβ_42_ levels, providing direct evidence that this *SHARPIN* variant is associated with late-onset AD (Asanomi et al., 2024).

In contrast to AD, the applications of CRISPR-Cas9 in managing PD are limited. This genome editing technique targets several genes associated with PD, including *SNCA*, *LRRK2*, *PRKN*, *PARK7*, and *PINK1* (Mansour and El-Khatib, 2023). CRISPR-Cas9 has been applied to directly delete the *SNCA*^A53T^ variant, leading to a reduction of α-synuclein, which mitigates dopaminergic neurodegeneration and alleviates symptoms in a PD mouse model (Yoon et al., 2022). In contrast, Han et al. (2024) used the CRISPR-Cas9 technique to develop *PRKN*-depleted monkey models to elucidate the potential clinical applications of this genome editing method. The *PRKN* knockout monkey model demonstrates an increase in phosphorylated parkin and a reduction in accumulated α-synuclein. Phosphorylated parkin is a critical target for managing PD, as it plays a significant role in neuroprotection. The complex and poorly understood pathogenic mechanisms underlying PD suggest that genome editing may not consistently yield beneficial outcomes. In a study conducted by Ahfeldt et al. (2020), the research team used isogenic human pluripotent stem cell lines to knock out several genes associated with PD, specifically *PRKN*, *PARK7*, and *PARK9*. Following these genetic modifications, increased levels of oxidative stress were observed across all PD gene knockout cell lines. Notably, the *PRKN*-deleted cells exhibited a heightened rate of death among nigral dopaminergic neurons during differentiation.

The management of HD via CRISPR-Cas9 has garnered significant attention from various research groups (Alkanli et al., 2023). In 2017, Yang et al. used the CRISPR-Cas9 technique to mitigate neurotoxicity in a murine model of HD. Given that the knockout of HTT results in embryonic lethality in mice, Yang et al. used this approach to permanently suppress endogenous mutant *HTT* (*mHTT*) expression in HD140Q-knockin mice. This was achieved through the direct injection of adeno-associated virus (AAV)-CMV-Cas9 and AAV-HTT-gRNA into the striatal region, which led to a reduction in HTT aggregates and a decrease in early neuropathological changes. The production of mutant proteins from *mHTT* contributes to the increase in neuron inclusions and degeneration of neurons in the striatum; therefore, the elimination of *mHTT* may represent a viable strategy for managing HD. In 2019, Ekman et al. (2019) injected AAV-gRNA into the striatum of the R6/2 mouse model, specifically targeting the disruption of *mHTT* expression through the introduction of frameshift-inducing insertions and deletions. The CRISPR-Cas9-mediated disruption of *mHTT* in these mice resulted in nearly a 50% reduction in neuronal inclusions, as well as improvements in motor deficits and an extension of lifespan. These findings suggest that the CRISPR-Cas9 technique is effective for targeting *mHTT* disruption; however, further investigation into potential off-target effects and the safety of AAV vectors is warranted. Replacing the expanded CAG repeat regions in the *HTT* gene with normal CAG repeats may present a promising strategy for managing HD. This approach involves the intracranial or intravenous injection of AAV vectors carrying Cas9 and gRNA that specifically target *mHTT*, along with donor DNA containing normal CAG repeats, in a porcine model. This intervention has been shown to alleviate the neurotoxicity associated with *mHTT* and mitigate neurological symptoms (Yan et al., 2023). Furthermore, a research team from China has recently developed an innovative CRISPR delivery system aimed at enhancing specificity for neuronal cells, thereby providing a novel approach for the treatment of HD (Ling et al., 2025). This delivery system, referred to as the Ribonucleoprotein Delivery System, is designed to selectively deliver Cas9 and gRNA into dendritic cells, neurons, and other relevant cell types. The Ribonucleoprotein Delivery System demonstrates significant efficiency in editing the *HTT* gene in induced pluripotent stem cell-derived neurons from patients with HD, potentially facilitating the advancement of CRISPR-Cas9 therapy *in vivo*.

## Current Status of Clinical Translation and Application

The ultimate goal of elucidating Cu metabolism and the process of cuproptosis is to facilitate the transition from basic research to clinical application, particularly in the treatment of NDDs. In this section, we summarize the development of chelators in both preclinical and clinical studies (**Additional Table 2** and **Box 1**).

**Additional Table 2:** Clinical trials of Cu chelators for the treatment of neurodegenerative diseases

Chelator	Disease	Origin of patients	Clinical trial/year	Outcome	Enrolled subjects	Reference/Clinical trial registration
CQ	AD	Australia	Phase II, 2003	250 mg of PBT2 reduced plasma Aβ42 levels in individuals with AD.	36	Ritchie et al., 2003
PBT2	AD	Sweden	Phase II, 2008	250 mg of PBT2 reduced CSF Aβ42 levels in individuals with AD.	74	Lannfelt et al., 2008
PBT2	AD	Sweden and Australia	Phase II, 2010	250 mg of PBT2 reduced CSF Aβ42 levels and alleviated cognitive impairment in individuals with AD.	78	Faux et al., 2010
PBT2	AD	Australia and Sweden	Phase II, 2008	No results posted.	80	ClinicalTrials.gov ID: NCT00471211
PBT2	HD	United States and Australia	Phase II, 2016	250 mg of PBT2 alleviated HD-symptoms and reduce motor impairment function in these patients.	109	Huntington Study Group Reach, 2015; ClinicalTrials.gov ID: NCT01590888
Curcumin	AD	China	Phase II, 2006	No results posted.	36	ClinicalTrials.gov ID: NCT00164749
Curcumin	AD	United States	Phase II, 2007	No results posted.	33	ClinicalTrials.gov ID: NCT00099710
Curcumin	AD	India	Phase II, 2010	No results posted.	26	ClinicalTrials.gov ID: NCT01001637
EGCG	AD	Spain	Phase II, 2023	EGCG slowed cognitive decline and improved brain connectivity.	129	ClinicalTrials.gov ID: NCT03978052
EGCG	AD	Germany	Phase II, 2015	No results posted.	21	ClinicalTrials.gov ID: NCT00951834
EGCG	PD	China	Phase II, 2009	No results posted.	480	ClinicalTrials.gov IDs: NCT00461942
EGCG	HD	Germany	Phase II, 2015	No results posted.	54	ClinicalTrials.gov IDs: NCT01357681
LA	AD	United States	Phase II, 2014	LA plus ω-3 fatty acids improve cognitive function and reduce inflammation, lipid dysregulation, and alleviate insulin resistance in patients with AD.	67	ClinicalTrials.gov IDs: NCT01058941
LA	AD	United States	Phase II, 2007	LA plus ω-3 fatty acids reduce oxidative levels and slow cognitive decline in patients with AD.	39	ClinicalTrials.gov IDs: NCT00090402

AD: Alzheimer's disease; Aβ_42_: amyloid-beta 42; CQ: clioquinol; CSF: cerebrospinal fluid; EGCG: epigallocatechin gallate; HD: Huntington's disease; LA: α-lipoic acid; PD: Parkinson's disease; PTB: 5,7-dichloro-2-[(dimethylamino) methyl]-8-hydroxyquinoline.

Among these chelators, clioquinol (CQ) and PBT2 have been evaluated in several clinical studies related to AD and HD. In 2003, a pilot phase II clinical trial involving 36 participants in Australia demonstrated that CQ significantly reduced plasma Aβ_42_ levels in individuals with AD. This Cu chelator was found to have an acceptable tolerability profile (Ritchie et al., 2003). In 2008, PBT2 underwent a phase II, double-blind, randomized, placebo-controlled trial in Europe. A total of 74 patients were randomly enrolled in the study, and no severe side effects were reported. Furthermore, the level of Aβ_42_ in CSF was significantly decreased in patients administered higher doses of PBT2 (up to 250 mg) (Lannfelt et al., 2008). In the same year, an interventional phase II clinical trial was conducted in which two doses of PBT2 were administered over 12 weeks to patients with early AD, compared with a placebo control group (ClinicalTrials.gov ID: NCT00471211). In HD, a randomized, double-blind, placebo-controlled study was conducted in the United States and Australia to evaluate the safety, tolerability, and efficacy of PBT2 in patients with early and mild stages of the disease. A dosage of 250 mg of PBT2 was found to alleviate HD symptoms and reduce motor impairment function in these patients (ClinicalTrials.gov ID: NCT01590888).

Curcumin has demonstrated considerable Cu chelation properties, suggesting its potential utility in reducing Cu toxicity (Maghool et al., 2023). In a completed phase II clinical trial, the efficacy and safety of two daily doses of curcumin (1 or 4 g), combined with 120 mg of ginkgo leaf extract, were evaluated in 36 human subjects (ClinicalTrials.gov ID: NCT00164749). Although curcumin was well tolerated, this initial pilot study did not demonstrate clinical efficacy in cognitive improvement. The limited sample size and short duration of the trial may have constrained the ability to evaluate the therapeutic effects of curcumin in AD (Baum et al., 2008). Additionally, two other recorded clinical trials investigating curcumin did not demonstrate therapeutic effects in patients with AD (ClinicalTrials.gov IDs: NCT00099710, NCT01001637).

Another Cu chelator, EGCG, has demonstrated potential neuroprotective effects and is capable of binding to the β-sheet of Aβ, thereby remodeling the Aβ conformation and reducing the toxicity of aggregated Aβ peptides (Tavanti et al., 2020). Two clinical trials were completed in Spain and Germany, identified by ClinicalTrials.gov IDs NCT03978052 and NCT00951834, respectively. The NCT03978052 study enrolled 129 participants to evaluate the beneficial effects of EGCG on patients with early AD. Compared with the placebo group, administration of EGCG slowed cognitive decline and improved brain connectivity; both groups, including placebo and EGCG participants, also underwent lifestyle interventions involving physical and cognitive training. The NCT00951834 trial enrolled 21 participants; however, the final results regarding the effects of EGCG in patients with AD were not reported by the researchers. Therefore, further investigation is required to determine the therapeutic efficacy of this chelator in patients with AD. Additionally, two phase II clinical trials of EGCG were conducted in China and Germany to investigate its therapeutic effects and safety in PD and HD, enrolling 480 and 54 participants, respectively (ClinicalTrials.gov IDs: NCT00461942 and NCT01357681). However, no publications or results from these trials have been identified.

Two clinical trials have been conducted to investigate the therapeutic effects of LA in slowing the progression of AD. The NCT01058941 trial enrolled 67 participants and compared cognitive and memory changes over 18 months between a placebo group and an intervention group receiving 600 mg of LA per day combined with 3 g of ω-3 (omega-3) fatty acids daily. Patients with AD in the intervention group demonstrated improved cognitive function, accompanied by reductions in inflammation, lipid dysregulation, and insulin resistance. Another clinical trial (NCT00090402) investigated the effects of ω-3 fatty acids and LA on the progression of AD by assessing oxidative markers in blood and urine samples. In this study, 39 patients with AD were divided into three groups: placebo, 3 g of ω-3 fatty acids alone, and 3 g of ω-3 fatty acids combined with 600 mg of LA. After 12 months of intervention, the combination of ω-3 fatty acids and LA was found to reduce oxidative marker levels and slow cognitive decline in patients with AD. However, given the limited sample size of 39 participants, further research is necessary to confirm the therapeutic efficacy of LA combined with ω-3 fatty acids.

According to the most recent records from the Food and Drug Administration, no Cu chelators, ionophores, or specific cuproptosis-related modulators have been approved for the treatment of AD, PD, HD, ALS, or prion diseases. While approvals have been granted for other Cu-related conditions, such as Wilson disease treated with trientine and tetrathiomolybdate, no such approvals currently exist for NDDs (Kamlin et al., 2024; Kirk et al., 2024).

## Limitations

Despite the growing body of research on the dysregulation of Cu metabolism and cuproptosis in NDDs, several limitations must be acknowledged when interpreting the findings presented in this review. These challenges are primarily attributable to both the current state of the literature and the intricate nature of metal homeostasis within the central nervous system. The existing literature exhibits considerable heterogeneity in study designs, methodologies, and experimental models. The studies reviewed utilize a diverse array of animal models and *in vitro* systems, which complicates the direct comparison of results and restricts the generalizability of conclusions. Furthermore, a substantial portion of the available data originates from preclinical studies, with a relatively small number of investigations conducted involving human subjects. This limited availability of robust human data constrains the translation of findings and highlights the necessity for well-designed clinical studies.

The complexity of Cu homeostasis presents significant analytical challenges. Accurately quantifying Cu species, their fluxes, and their compartmentalization *in vivo* is demanding in animal models, often leading to varying and occasionally conflicting results across different studies. Additionally, the mechanisms and markers associated with cuproptosis are still under investigation, and there is currently an absence of standardized methods for its assessment. Cuproptosis remains a relatively novel area of research. This lack of consensus impedes the reproducibility and interpretation of experimental findings within this emerging field.

Confounding factors, including age, comorbid conditions, genetic variability, and environmental exposures, often influence clinical studies of neurodegeneration, thereby complicating the establishment of definitive causal relationships. Furthermore, given that cuproptosis and its significance in the pathology of NDDs represent a rapidly evolving field of research, there is a notable scarcity of long-term and longitudinal studies. Despite these limitations, the review highlights the substantial progress made in elucidating the roles of Cu dysregulation and cuproptosis in the therapeutic approaches for NDDs. It will be crucial to address the identified challenges in future research.

## Conclusion and Perspectives

NDDs characterized by memory loss and progressive cognitive impairment, including AD, PD, HD, ALS, and prion diseases, have become a significant threat to public health. Although the pathogenesis of NDDs has not been fully determined, several risk factors have been elucidated. Among these factors, the pathogenic molecular mechanisms of Cu have drawn attention in recent decades; this is likely due to the fact that imbalances in Cu metabolism contribute to neuronal cell death, neuroinflammation, oxidative stress, and pathogenic protein aggregation.

Unlike other articles that focus solely on one or two NDDs, this review adopts a broader perspective by elucidating the pathogenic roles of Cu dysregulation across numerous NDDs and discussing potential therapeutic strategies to manage these conditions. We also integrated and highlighted recently published articles that decipher the association between cuproptosis and the progression of NDDs, even though this novel programmed cell death mechanism has not been fully understood in these diseases, such as the limited publications regarding the detrimental effects of cuproptosis in HD and ALS. Additionally, we created a box to highlight critical clinical trials demonstrating the therapeutic potential of Cu chelators for alleviating symptoms of AD, PD, and HD (**Box 1**).

Cu plays critical roles in various physiological processes, ranging from macroscopic to microscopic functions, though its effects can be both beneficial and detrimental. On one hand, Cu is an essential cofactor for numerous enzymes; on the other hand, dysregulation of Cu can lead to oxidative stress and cell death. In the brain, Cu metabolism affects protein aggregation and neuroinflammation. Thus, orchestrated Cu metabolism and concentration are required to ensure its proper functioning. Moreover, the novel programmed cell death mechanism, cuproptosis, may offer new insights into the pathogenic roles of Cu in NDDs.

The transition from bench research to clinical application presents significant challenges; therefore, identifying therapeutic targets is fundamental for managing NDDs. With advancements in bioinformatics and machine learning models, numerous studies have identified CRGs as potential critical biomarkers for diagnosis and as therapeutic targets for treatment (Lai et al., 2022; Zhang et al., 2022a). Particularly in AD and PD, various CRGs have been identified, exhibiting distinct expression patterns among patients. However, it is important to note that these CRGs may overlap in both diseases, and the diversity of patients’ immune infiltrations should not be overlooked. A comprehensive understanding of the relationship between CRGs and the progression of NDDs necessitates a multifaceted approach, including *in vitro* experiments, *in vivo* studies, and the establishment of large clinical cohorts.

In addition to cuproptosis, pathways and enzymes associated with the dysregulation of Cu metabolism represent promising therapeutic targets for NDDs. Several studies have shown that Cu removal is beneficial in treating NDDs (Wang et al., 2018; Chen et al., 2023, 2025). However, there are still many obstacles to the clinical use of Cu chelators. For instance, CQ has been discontinued from further development due to increased contamination during large-scale synthesis and its inability to produce the desired results in phase III clinical trials. Additionally, many chelator-based therapies have undesirable side effects. Administration of a high dose of EGCG resulted in hepatic necrosis in an animal model. Due to the constraints of bioavailability and the potential for adverse reactions associated with conventional metal chelators, there is a growing interest in novel compounds that possess properties beyond chelating activity. There is a need to develop metal chelators with high bioavailability and biocompatibility to effectively target NDDs. However, many aspects still need to be determined, such as optimal dosage and molecular mechanisms.

Lastly, with the progressive advancement of CRISPR-Cas9 genome editing techniques, novel approaches have been proposed for the treatment of NDDs, as several genetic mutations associated with these disorders have been identified. Given the increasing severity and complexity of NDDs in the context of global aging and the challenges associated with their treatment, it is imperative not to underestimate their prevalence and impact. This review aims to elucidate the pathogenic roles of Cu metabolism and Cu-induced programmed cell death, referred to as cuproptosis, in the progression of NDDs. Furthermore, we aspire to offer novel insights that may enhance understanding, management, and potential prevention of these diseases.

## Additional files:

***Additional Table 1:*** *Preclinical investigations of Cu chelators for the treatment of neurodegenerative diseases.*

***Additional Table 2:*** *Clinical trials of Cu chelators for the treatment of neurodegenerative diseasess.*

## Data Availability

*All relevant data are within the paper and its Additional files.*
